# Efficacy and Safety of Albumin in Critically Ill Adults: A Systematic Review

**DOI:** 10.1155/emmi/7434218

**Published:** 2026-07-20

**Authors:** Talía Gabriela Porras Suárez, Marta Gutiérrez Valencia, Luis Carlos Saiz, Juan Pedro Tirapu León, Aitor Ansotegui Hernández, Amaia Egüés Lugea, Leire Leache Alegría, Juan Erviti

**Affiliations:** ^1^ Department of Health Sciences, Public University of Navarra, Pamplona, Navarra, Spain, unavarra.es; ^2^ Innovation and Organization Unit, SNS-O, Pamplona, Navarra, Spain; ^3^ Intensive Medicine Service, University Hospital of Navarra, Pamplona, Navarra, Spain, cun.es; ^4^ Pharmaceutical Benefit Management Service, Pharmacy and Benefits Subdirectorate, SNS-O, Pamplona, Navarra, Spain

**Keywords:** albumin, effectiveness, ICU, meta-analysis, safety, systematic review

## Abstract

**Introduction:**

Albumin, the most abundant blood protein, is widely used as a plasma expander in critically ill patients.

**Objective:**

To evaluate the efficacy and safety of albumin compared to other alternatives in the prevention and treatment of complications in critically ill adults, considering different comparators and clinical situations.

**Methods:**

A systematic review was conducted, including randomized controlled trials (RCTs) comparing albumin with crystalloids, other colloids, or standard care without albumin. Primary outcomes were total mortality, blood transfusion requirement, and serious adverse events. The search was performed in MEDLINE, Embase, and Cochrane Central. Meta‐analysis was conducted using the Mantel–Haenszel method with a random‐effects model, and heterogeneity was assessed with the I^2^ statistic. The risk of bias was evaluated using Cochrane RevMan 5.4, and the quality of evidence was assessed with the GRADE approach.

**Results:**

Thirty‐five RCTs involving 13,975 critically ill adults were included. Mortality was 23.8% (1327/5586) with albumin and 24.2% (1361/5618). Compared with colloids, mortality was 21.9% (83/379) versus 24.4% (113/463). When compared with standard care without albumin, mortality was higher with albumin: 15.8% (60/380) versus 9.2% (39/424). SAEs occurred in 37.6% (298/793) of albumin‐treated patients versus 33.5% (266/793) with crystalloids. Albumin required less transfused blood volume than colloids.

**Conclusions:**

Albumin showed no benefit over crystalloids, colloids, or standard care without albumin in reducing mortality. It resulted in less blood transfusions compared to colloids, but serious adverse events were higher than with crystalloids.

## 1. Introduction

Albumin (ALB) is responsible for maintaining oncotic pressure and for transporting endogenous and exogenous substances [1]. As a blood‐derived product, its availability is limited, and its administration should be evidence‐based. Beyond its oncotic properties, ALB exhibits antioxidant, buffering, and immunomodulatory effects that could theoretically improve outcomes in critically ill patients [2–4].

In critical illness, ALB contributes to endothelial stabilization and maintenance of the glycocalyx, reducing capillary leakage and fluid extravasation [5]. It also supports acid–base balance and drug transport, particularly for antibiotics in sepsis [6]. These pleiotropic functions have led to the hypothesis that ALB might improve hemodynamic stability and clinical outcomes in severe conditions such as sepsis, trauma, burns, or surgery [7, 8].

However, the clinical effectiveness and safety of ALB remain controversial. Several systematic reviews and meta‐analyses have provided inconsistent results, partly due to heterogeneity in study populations, ALB concentrations, and comparators [9–11]. In addition, concerns about adverse effects and high cost have limited its widespread use.

Therefore, this systematic review aimed to assess the efficacy and safety of ALB versus other therapeutic alternatives in critically ill adults, across diverse clinical contexts and comparators, and to provide an updated synthesis of the available evidence.

The objective of this review is to evaluate the efficacy and safety of ALB versus other alternatives in the prevention and treatment of complications in critically ill adults, considering different comparators and clinical conditions.

## 2. Methods

This systematic review was designed in accordance with the criteria of the Preferred Reporting Items for Systematic Reviews and Meta‐Analyses (PRISMA) [11]. We registered the protocol in PROSPERO, an international prospective registry of systematic reviews (registration ID = CRD42024512102).

### 2.1. Research Question

To assess the efficacy and safety of ALB compared to other alternatives in critically ill patients, the review was designed to analyze three different populations of critically ill patients (adults, children, and elective surgery). This article reports results on critical adults. The PICO question is described in detail in Appendix 1 (Supporting Information) of this review. Only randomized controlled trials (RCTs) were considered.

Critically ill patients were defined as those suffering from a life‐threatening clinical condition that could cause death or serious harm. Patients meeting the above description were included when the study characterized them as critically ill or when they were admitted to intensive care units (ICUs) or emergency departments (EDs).

This included shock, neurological disease, trauma, edema due to hypoalbuminemia, surgery, and burns, among others.

According to the International Council for Harmonization of Technical Requirements for Pharmaceuticals for Human Use (ICH) [12], serious adverse events (SAEs) were defined as any event that results in death, is life‐threatening, requires hospitalization or prolongation of existing hospitalization, results in persistent or significant disability, or is a congenital anomaly or birth defect [13]. Mortality and SAE outcomes were extracted according to the follow‐up period reported in each trial. This variability in follow‐up duration is acknowledged as a source of heterogeneity in the pooled analysis.

The intervention consisted of ALB (administered at any dose, time interval, and for any duration). The following comparators were considered: (1) crystalloids, (2) colloids other than ALB, (3) crystalloids in combination with colloids, and (4) standard care without ALB.

The primary outcomes were overall mortality, volume of blood transfusion requirement, and SAEs. Secondary outcomes included organic dysfunction, volume of blood loss, in‐hospital and ICU length of stay, total adverse events, hospital and ICU readmission, total fluid volume, and fluid balance.

Mortality was analyzed according to the follow‐up duration reported in each RCT and was temporally categorized due to substantial variability across studies (28 days–36 months) to improve clinical interpretability and reduce heterogeneity. Short‐term mortality was defined as death within 30 days, including 28‐day and in‐hospital mortality when applicable, primarily reflecting the acute effects of the intervention. Long‐term mortality was defined as death occurring beyond 90 days, including 90‐day, 6‐month, and > 6‐month mortality, which are more likely influenced by comorbidities and postacute factors. The primary outcome was all‐cause mortality at the longest available follow‐up in each study, which maximizes data inclusion but introduces clinical and methodological heterogeneity due to differences in follow‐up duration.

### 2.2. Search Methods

A literature search was performed in MEDLINE (access via PubMed), EMBASE, and Cochrane Central on February 7, 2024. Studies were included regardless of language or date of publication. Clinicaltrials.gov was also searched to identify RCTs meeting the planned criteria. Full search strategy is available in Appendix 2 (Supporting Information).

### 2.3. Study Selection

A study screening was carried out with the Rayyan software [14], and data extraction was peer‐reviewed. Data analysis, synthesis, and risk of bias assessment were performed with RevMan 5.4 software according to Cochrane methodology [15, 16].

### 2.4. Data Analysis

Dichotomous outcomes corresponded to the proportion of participants who experienced at least one event. They were expressed as relative risk (RR) with their respective 95% confidence interval (95% CI). In the case of continuous variables, the mean difference (MD) was reported with the corresponding 95% CI.

We considered an *I* ^2^ > 60% as indicative of substantial heterogeneity. The results were meta‐analyzed according to the Mantel–Haenszel method, using a random‐effects model. The certainty of the evidence was evaluated according to GRADE methodology [17].

### 2.5. Subgroup and Sensitivity Analyses

For the primary outcomes, subgroup analyses were performed according to specific comparators and clinical conditions. A sensitivity analysis restricted to studies with low risk of bias was also carried out.

Due to variability in ALB concentrations and dosing regimens, as well as inconsistent reporting across studies, a quantitative subgroup analysis based on these parameters was not feasible. We conducted an additional sensitivity analysis excluding studies with follow‐up beyond 90 days.

### 2.6. Ethical Approval

The data for this systematic review were obtained from published RCTs and thereby ethical approval was not required.

## 3. Results

**FIGURE 1:**
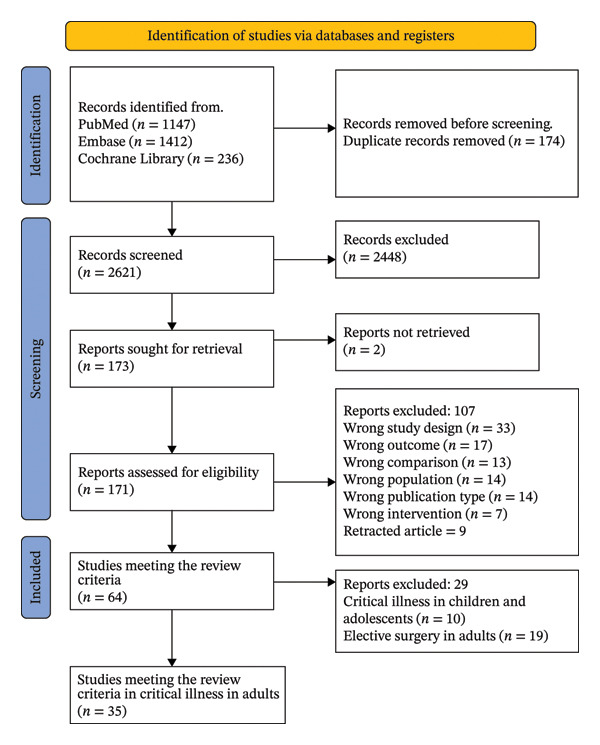
PRISMA flowchart showing the study selection and exclusion process.

The search identified 2795 articles. After a first title/abstract screening, 171 reports were reviewed in full text. A total of 64 RCTs met the inclusion criteria, of which 35 corresponded to critical adults. The results of the selection process are presented in Figure 1.

Included studies were published between 1975 and 2023, had a sample size between 10 and 7000 patients, and globally included 13,975 participants. As many as 35 studies included critically ill adults with different clinical conditions. A total of 15 studies used crystalloids as a comparator, while 11 used different types of colloids and 12 used standard care without ALB. Some studies had more than two arms, presenting more than one comparator. The duration of follow‐up ranged from 28 days to 36 months, and the mean age of the participants was 55 years, ranging from 18 to 70 years, with a standard deviation of 38.5 years. The main characteristics of the included studies are summarized in Table 1, and the summary of results on the primary and secondary outcomes is presented in Tables 2 and 3.

**TABLE 1:** Characteristics of the studies.

**Author, year**	**Center**	**Blinding**	**Participants (no.)**	**Age (years)**	**Sex (males)**	**Diagnosis or clinical features**
**Albumin vs. crystalloids**	**Critical adults**					

Bisgaard J, 2020	UC	Double‐blind	89	I: 68 [62–71] C: 65 [59–72]	I: 23 (70) C: 19 (63)	Adults scheduled for elective major upper gastrointestinal cancer surgery
Caironi 2014	MC	Open‐label	1818	I: median 70 C: median 60	I: 543 (60.1%) C: 550 (60.6%)	Severe sepsis
Cortegiani A, 2021	MC	Open‐label	304 (139 albumin; 165 crystalloids)	I: 63.2 ± 13.8 C: 63.1 ± 13.5	I: 25.1 ± 4.0 C: 25.4 ± 4	Cirrhosis and sepsis
Dubois MJ, 2006	UC	Open‐label	The two groups of 50 patients (100)	I: 63.0–14.3 C: 65.2–13.7	I: median 31 C: median 30	Critical patients with hypoalbuminemia
Ernest D, 1999	UC	Unblinded	18	I: mean 51, SD: 21 C: mean 55, SD 17	I: 5 C: 6	Septic, critically ill patients
Goslinga H, 1992	UC	A blind control group is not possible, either for the patient or for the medical staff	NR	NR	NR	Ischemic stroke
Pesonen E, 2022	UC	Double‐blind	1407	I: 65 (10) C: 65 (10)	I: 549 (79) C: 542 (78)	Patients undergoing on‐pump coronary artery bypass grafting
Maiwall, 2022	UC	Open‐label	100: Group A: 50 and Group B: 50	I: 50.58 ± 9.87 C: 47.28 ± 11.29	I: 44 (88) C: 44 (88)	Cirrhosis and sepsis
Oczkowski SJW, 2018	UC	Single‐blinded	45	I: 61.71 (17.2) C: 64.70 (15.2)	I: 13 (54.2) C: 16 (76.2)	(Adults) with hypoalbuminemia
Park 2019	UC	Double‐blind	360	I: 62 (51–70) C: 61 (52–70)	I: 96 (53%) C: 90 (50%)	Cancer patients with severe sepsis or septic shock
Philips 2022	UC	Open‐label trial	308 patients included (154 human albumin, 154 normal saline)	I: 49.4 ± 12.1 C: 48.2 ± 10.6	I: 123 (79.8%) C: 131 (85.1%)	Cirrhosis
SAFE 2004 (Finfer 2006 and 2011, Myburgh 2007, and Bellomo 2009)	MC	Double‐blind	Seven thousand patients were randomized, 3499 to the albumin group and 3501 to the saline group; both groups had similar baseline characteristics	I: 58.6 ± 19.1 C: 58.5 ± 18.7	I: 1424 (40.7) C: 1376 (39.3)	Cirrhosis and sepsis
Gondos T, 2010	MC	NR	200	I: 59–13 C: 60–15/58–16/53–16	I: 21/29 C: 26/24/30/20/24/26	Postoperative hypovolemic patients
Veneman TF, 2004	UC	NR	63	I: 72 C: 67, 68, 71	I: 8 C: 10, 9, 9	Severely‐ill patients with hypoalbuminemia: sepsis, postsurgical patients with SIRS
Rackow 1983	UC	NR	26	I: median 82 C: median 80 (H) 72 (S)	I: 68% C: 78% (H) 50% (S)	Shock hypovolemic

**Albumin vs. colloids**	**Critical adults**					

Waxman 1988	NR	NR	12 (cross‐over trial)	Mean 42 years (range: 16–77)	8 (67%)	Patients selected for the study were those who suffered thermal burns of greater than 25% total body surface and who gave informed consent
Dolecek M, 2009	UC	NR	56	I: 47 (19–81) C: 43 (23–67)	I: 26/4 C: 22/4	Patients with severe sepsis
Falk JL, 1988	UC	NR	12	Median 78 years (range: 54–90)	10 (83%)	Septic shock
Lazrove S, 1980	UC	NR	10 (cross‐over)	Range: 51–77	5 (50%)	Acutely ill postoperative patients were selected for study from the surgical ICV at LAC Harbor UCLA Medical Center
Rackow 1989	UC	NR	20	Range: 35–90 years	15 (75%)	Severe sepsis
Stockwell 1992	UC	NR	475	I: 60 years (18–86) C: 64 years (18–88)	I: 159 (70%) C: 162 (65%)	11% trauma, 30% postoperative, 29% general medical, 13% respiratory failure, and 17% cardiac
Suzuki 2020	UC	Open trial	50	I: 65 (60–71) C: 70 (62–75)	I: 70 (62–75) C: 18 (72%)	Liver, bile duct, or pancreatic cancer
Abdelmotaal AM, 2024	UC	Open trial	52	I: 37.2 ± 10.2 C: 36.8 ± 12.2	I: 14 (53.8%) C: 15 (57.7%)	Massive burn patients
Gondos T, 2010	MC	NR	200	I: 59–13 C: 60–15/58–16/53–16	I: 21/29 C: 26/24/30/20/24/26	Postoperative hypovolemic patients
Veneman TF, 2004	UC	NR	63	I:72 C:67,68,71	I:8 C:10,9,9	Severely‐ill patients with hypoalbuminemia: sepsis, postsurgical patients with SIRS
Rackow 1983	UC	NR	26	I: median 82 C: median 80 (H) 72 (S)	I: 68% C: 78% (H) 50% (S)	Hypovolemic shock

**Albumin vs. standard care without albumin.**	**Critical adults**					

Goodwin 1983	UC	NR	79	I: 28 ± 7 C: 28 ± 8	NR	Hemodynamic response and on lung water following thermal injury
Cooper 2005	MC	NR	42 (the trial was suspended in June 2001 owing to slow enrollment, when 42 of 90 (the denominator was the target) patients had been randomly assigned)	I: 36 (24–45) C: 31 (25–39)	I: 15 (79%) C: 21 (91%)	Cutaneous thermal burns were assessed for possible enrollment
Jelenko C, 1978	UC	NR	19	I: 34.0 + −5.3 C: 52.4 + −12.7/47.3 + −5.6	NR	Patients admitted to the burn service
Lucas CE, 1980	UC	NR	94	I: 34.1 + 12.8 C: 33.1 ∗ 14.9	NR	Injured patients
Foley EF,1990	UC	NR	40	I: 63.2 ± 4.2 C: 66.4 ± 3.4	I: 55 C: 68	Hypoalbuminemic (serum albumin: 25 g/L [2.5 g/dL]), critically ill patients
Golub R, 1994	UC	Unblinded	262 randomized (219 included + 43 excluded)	I: 69.5 + −14.8″ C: 72.4 + 16.8	I: 40–60 C: 49–51	Admission circulating albumin concentrations of < 3.0 g/dL (< 30 g/L).
Grundmann R, 1986	UC	NR	161	NR	NR	Postoperative period in the surgical intensive care unit
Johnson SD, 1979	UC	NR	94	NR	NR	Oligemic shock
Weaver DW, 1978	UC	NR	52	NR	NR	Multiple transfusions, surviving at least 24 h
Martin 2005	MC	Double‐blinded	40	I: 46.4 (18.0) C: 48.9 (21.6)	I: 50 C: 45	Hypoproteinemic patients with acute lung injury
Lowe RJ, 1977	UC	Open‐label	137	NR	NR	Patients who required laparotomy for acute abdominal trauma
Lowe RJ, 1979	UC	Open‐label	141	I: 27.6 + −9.6 (1 + 5D) C: 32.3 + −12.5	I: 73 (89%) C: 52 (94%)	Patients with severe sepsis

**TABLE 2:** Summary of the results on the primary outcomes.

Comparison	Outcome	No. of studies (participants)	Albumin n/N (%)	Control n/N (%)	Certainty of evidence (GRADE)
Albumin vs. crystalloids	Mortality	10 (11,204)	1327/5586 (23.8%)	1361/5618 (24.2%)	Moderate (⨁⨁⨁◯)^a^
	Transfusion requirement (L)	5 (4247)	NA	NA	Very low (⨁◯◯◯)^abc^
	Serious adverse events	3 (1586)	298/793 (37.6%)	266/793 (33.5%)	Low (⨁⨁◯◯)^ab^

Albumin vs. colloids	Mortality	8 (842)	83/379 (21.9%)	113/463 (24.4%)	Very low (⨁◯◯◯)^ab^
	Transfusion requirement (L)	3 (57)	NA	NA	Low (⨁⨁◯◯)^bc^
	Serious adverse events	3 (100)	7/52 (13.5%)	8/48 (16.7%)	Very low (⨁◯◯◯)^ab^

Albumin vs. standard care without albumin	Mortality	8 (804)	60/380 (15.8%)	39/424 (9.2%)	Low (⨁⨁◯◯)^ab^
	Transfusion requirement (L)	5 (590)	NA	NA	Moderate (⨁⨁⨁◯)^a^
	Serious adverse events	2 (38)	15/99 (15.2%)	23/102 (22.5%)	Very low (⨁◯◯◯)^ab^

Abbreviations: MD, mean difference; NA, not applicable; NNH, number needed to harm (NNH).

^a^One‐level decrease due to study design limitations (risk of bias).

^b^One‐level/two‐level decrease due to imprecision.

^c^One‐level decrease due to inconsistency.

**TABLE 3:** Summary of secondary outcomes.

Comparison	Outcome	No. of studies (participants)	Albumin	Control	Heterogeneity (*I* ^2^)
Albumin vs. crystalloids	Total adverse events	2 (282)	18/137 (13.13%)	5/145 (3.44%)	0%
	Length of hospital stay (days)	7	MD: −0.33 (95% CI: −0.89−0.22)	—	90%
	Organ dysfunction	— (8978)	516/4472 (11.5%)	506/4506 (11.23%)	0%
	Length of ICU stay (days)	8	MD: −0.16 (95% CI: −0.61−0.29)	—	50%
	Fluid balance (L)	—	MD: −0.45 (95% CI: −1.29−0.39)	—	100%
	Duration of mechanical ventilation	3	MD: 0.00 (95% CI: −0.02−0.03)	—	0%
	Need for respiratory support	3 (7390)	2293/3679 (62.3%)	2349/3711 (63.3%)	0%
	Vasoconstrictor requirement	4 (1357)	296/680 (43.5%)	305/677 (45.0%)	0%

Albumin vs. colloids	Vasoconstrictor requirement	— (101)	22/45 (48.8%)	18/56 (32.1%)	78%
	SOFA score	—	MD: −1.00 (95% CI: −2.76−0.76)	—	—
	Duration of mechanical ventilation	2	MD: 2.74 (95% CI: −3.73−9.21)	—	40%
	Fluid balance (L)	5	MD: 0.07 (95% CI: −0.28−0.41)	—	60%
	Total volume infused (L)	7	MD: −0.04 (95% CI: −0.16−0.07)	—	80%
	Length of ICU stay (days)	2	MD: −2.30 (95% CI: −8.67−4.07)	—	80%
	Blood loss (L)	1	MD: 0.14 (95% CI: −0.03−0.30)	—	—

Albumin vs. standard care without albumin	Total adverse events	2 (261)	64/135 (47.4%)	56/126 (44.4%)	41%
	Need for respiratory support	4 (384)	97/175 (55.4%)	89/209 (42.5%)	0%
	Duration of mechanical ventilation	4	MD: 3.24 (95% CI: −0.20−6.68)	—	87%
	Total volume infused (L)	6	MD: 0.19 (95% CI: −1.52−1.90)	—	93%
	Length of ICU stay (days)	4	MD: 1.95 (95% CI: −1.09−5.00)	—	70%
	Length of hospital stay (days)	1	MD: 8.60 (95% CI: 2.56–14.64)	—	—

Abbreviations: MD, mean difference; NA, not applicable; NNH, number needed to harm.

**FIGURE 2:**
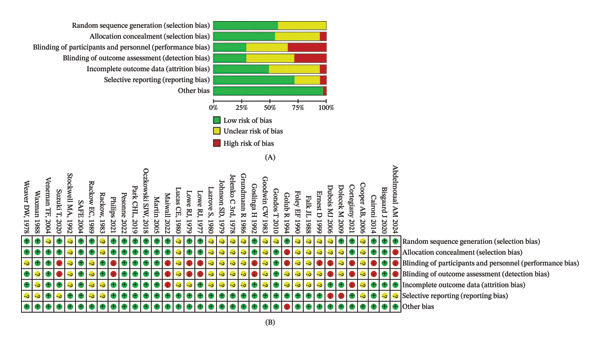
Summary of risk of bias. (A) Authors’ judgment for each risk of bias item presented as a percentage of the total included studies. (B) Authors’ judgment on each risk of bias item for each included study.

Regarding the risk of bias of included studies, 65% of the domains had a low risk of bias. Approximately 30% of studies presented an unclear risk of bias, and therefore, we should be cautious about the interpretation of results in these cases. Furthermore, although high risk of bias is less common, averaging 10%, its presence in critical areas, such as blinding of participants and personnel, may compromise the validity of some studies. The overall risk of bias was rated from low to unclear, though the lack of blinding might have influenced results in some outcomes (Figures 2A and B).

### 3.1. ALB vs. Crystalloids

**FIGURE 3:**
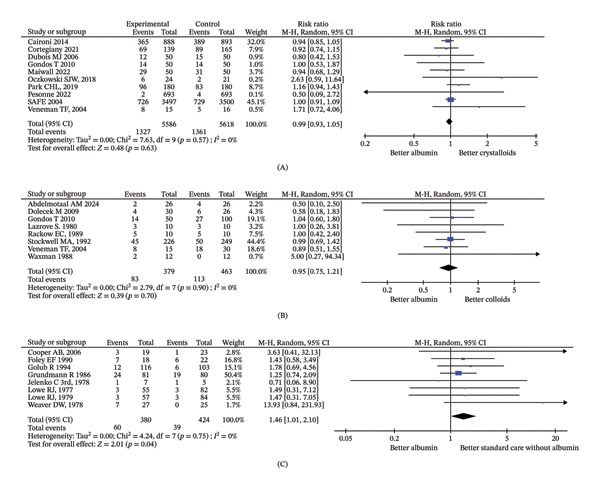
Mortality in critically ill adults, albumin vs. comparator: (A) crystalloids, (B) colloids, and (C) standard care without albumin.

As many as 15 studies compared the use of ALB versus crystalloids. The addition of albumin makes little or no difference in mortality (23.76% [1327/5586] vs. 24.23% [1361/5618]; 10 studies; *I* ^2^ = 0%; moderate certainty of evidence; Figure 3A). However, we are uncertain whether albumin alters the volume of blood required because the certainty of the evidence is very low (MD: −0.02 liters, 95% CI: −0.13 to 0.08; 5 studies; *I* ^2^ = 99%).

**FIGURE 4:**
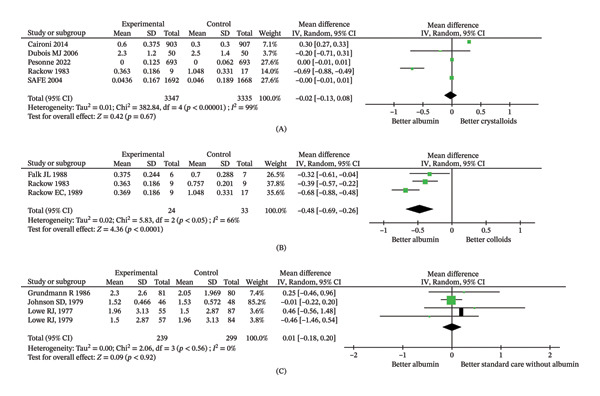
Blood requirement in critically ill adults, albumin vs. comparator: (A) crystalloids, (B) colloids, and (C) standard care without albumin.

No significant difference in blood requirement was seen between albumin and crystalloids, although heterogeneity between studies was extremely high (Figure 4A).

**FIGURE 5:**
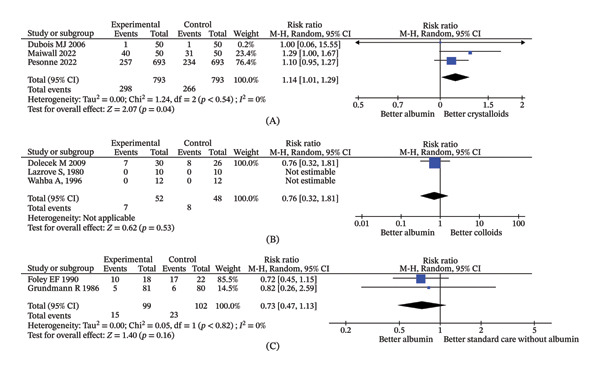
Serious adverse events in critically ill adults, albumin vs. comparator: (A) crystalloids, (B) colloids, and (C) standard care without albumin.

Albumin may increase the risk of SAEs compared to crystalloids (37.58% [298/793] vs. 33.54% [266/793]; 3 studies; *I* ^2^ = 0%; low certainty of evidence; Figure 5A).

The incidence of total adverse events was 3.8‐fold higher in the ALB group than in the control group, affecting 13.14% (18/137) and 3.45% (5/145) of patients, respectively, across two studies.

Likewise, no statistically significant difference was observed in the length of ICU stay (MD: −0.16 days; 95% CI: −0.61−0.29; 8 studies; (*I* ^2^) = 50%) or in fluid balance (MD: −0.45 liters; 95% CI: −1.29−0.39; (*I* ^2^) = 100%).

The subgroup analysis for mortality according to shock type shows consistent results in the septic shock, hypovolemic shock, and other subgroups (subgroup p‐test = 0.70). The meta‐analysis examined blood requirements (L) when comparing ALB versus crystalloids across three contexts: septic shock, hypovolemic shock, and others, showing a significant interaction between subgroups (*p* < 0.00001). In the “septic shock” subgroup, which included only one study, patients receiving ALB required significantly more blood, whereas in the hypovolemic shock subgroup, one study found that the crystalloid group required more blood. The “others” group showed more heterogeneous results.

### 3.2. ALB vs. Colloids

A total of 11 studies compared the use of ALB versus different colloids: hydroxyethyl starch (HES) (*n* = 7), pentastarch 10% (*n* = 2), and polygeline (*n* = 2). No significant difference in mortality was 21.9% (83/379) versus 24.4% (113/463), 8 studies, *I* ^2^ = 0%, very low certainty of evidence (Figure 3B). The use of albumin resulted in a lower volume of blood required compared to colloids (MD: −0.48 liters, 95% CI: −0.69–−0.26; 3 studies; *I* ^2^ = 66%); however, the certainty of the evidence was low.

Three studies provided information on SAEs. However, two of them had only 10 and 12 patients per group, respectively, and no SAEs were registered, 13.5% (7/52) vs. 16.6% (8/48) (Figure 5B). There was little or no difference between groups in the requirement of vasoconstrictors (48.89% [22/45] vs. 32.14% [18/56]; *I* ^2^ = 78%), SOFA score (MD: = 1.00, 95% CI: −2.76–0.76), or duration of mechanical ventilation (MD: 2.74 days, 95% CI: −3.73–9.21; 2 studies; *I* ^2^ = 40%). Similarly, the interventions may make little or no difference in fluid balance (MD: 0.07 liters, 95% CI: −0.28–0.41; 5 studies; *I* ^2^ = 60%), total volume infused (MD: −0.04 liters, 95% CI: −0.16–0.07; 7 studies; *I* ^2^ = 80%), or length of ICU stay (MD: −2.30 days, 95% CI: −8.67–4.07; 2 studies; *I* ^2^ = 80%).

Only one study [18] reported information on blood loss, and no difference was found between groups, MD: 0.14 L (95% CI: −0.03–0.30).

Subgroup analyses in both septic shock and other clinical contexts showed consistent results and did not demonstrate a significant difference in mortality.

For the outcome of SAEs, all three included studies enrolled patients with septic shock; therefore, no subgroup analysis was performed.

Regarding blood transfusion requirements, subgroup analyses showed consistent findings across groups, suggesting a potential benefit across different types of shock.

### 3.3. ALB vs. Standard Care Without Albumin

A total of 12 studies compared the use of albumin added to standard care versus standard care alone. The addition of albumin may increase mortality compared to standard care (15.79% [60/380] vs. 9.20% [39/424]; 8 studies; *I* ^2^ = 0%; low certainty of evidence).

The intervention makes little or no difference in blood requirement between groups (MD: 0.01 liters, 95% CI: −0.18–0.20; 4 studies; *I* ^2^ = 0%; moderate certainty of evidence). However, we are uncertain whether albumin alters the risk of SAEs because the certainty of the evidence is very low (15.15% [15/99] vs. 22.55% [23/102]; 2 studies; *I* ^2^ = 0%).

Only one small study reported information on the need for vasoconstrictors. No statistically significant difference was observed in the incidence of total adverse events (47.41% [64/135] vs. 44.44% [56/126]; 2 studies; *I* ^2^ = 41%). However, an increase in the need for respiratory support was found in the albumin group (55.42% [97/175] vs. 42.58% [89/209]; 4 studies; *I* ^2^ = 0%). The numerical increase in the duration of ventilator use in the albumin group did not reach statistical significance although the lower limit of the confidence interval approached the margin of effect (MD: 3.24 days, 95% CI: −0.20–6.68; 4 studies; *I* ^2^ = 87%).

No statistically significant difference was observed between groups in total volume infused (MD: 0.19 liters, 95% CI: −1.52–1.90; 6 studies; *I* ^2^ = 93%) or length of ICU stay (MD: 1.95 days, 95% CI: −1.09 to 5.00; 4 studies; *I* ^2^ = 70%). However, albumin use was associated with a longer duration of hospital stay (MD: 8.60 days, 95% CI: 2.56–14.64; 1 study [10]). This study also reported information on the risk of organic dysfunction, and no difference between groups was observed.

The subgroup analysis for mortality, including data from seven studies, suggests that the use of ALB may be more harmful in patients with hypovolemic shock than in other populations (test for subgroup differences *p* = 0.10). However, the estimate is imprecise, and further studies are required to confirm the effect across different populations (Tables 4 and 5).

**TABLE 4:** Subgroup analysis: mortality in critically ill adults.

Comparison	Subgroup	Albumin n/N (%)	Control n/N (%)	Subgroup *p* value	Interpretation
Albumin vs. crystalloids	Global	1327/5586 (23.8%)	1361/5618 (24.2%)	0.70	No difference
	Septic shock	≈ 24%	≈ 24%		Consistent
	Hypovolemic shock	≈ 24%	≈ 24%		Consistent
	Other	≈ 24%	≈ 24%		Consistent

Albumin vs. colloids	Global	83/379 (21.9%)	113/463 (24.4%)	NS	No difference
	Septic shock	≈ 22%	≈ 24%		Consistent
	Other	Similar	Similar		Consistent

Albumin vs. standard care without albumin	Global	60/380 (15.8%)	39/424 (9.2%)	0.10	Higher mortality
	Hypovolemic shock	≈ 16%	≈ 9%		Possible harm
	Other	Similar trend			Consistent

**TABLE 5:** Subgroup analysis: adverse events and transfusion.

Comparison	Outcome	Subgroup	Albumin n/N (%)	Control n/N (%)	Interpretation
Albumin vs. crystalloids	Serious adverse events	Global	298/793 (37.6%)	266/793 (33.5%)	Higher with albumin
	Transfusion	Septic shock	↑	↓	Single study
	Transfusion	Hypovolemic shock	↓	↑	Single study

Albumin vs. colloids	Serious adverse events	Global	7/52 (13.5%)	8/48 (16.7%)	No difference
	Transfusion	Global	↓	↑	Consistent benefit

Albumin vs. standard care without albumin	Serious adverse events	Global	15/99 (15.2%)	23/102 (22.5%)	No difference
	Respiratory support	Global	97/175 (55.4%)	89/209 (42.5%)	Higher with albumin

In the primary analysis, mortality rates were comparable between groups (23.8% vs. 24.2%), with no statistically significant differences and no observed heterogeneity (*I* ^2^ = 0%). After exclusion of studies with follow‐up longer than 90 days, mortality rates remained comparable between groups, with no evidence of a clinically or statistically meaningful difference. Although exact pooled percentages could not be derived due to variability in reporting across studies, the direction and magnitude of effect remained unchanged (Table 6).

**TABLE 6:** Sensitivity analysis excluding studies with follow‐up > 90 days.

Analysis model	Studies (n)	Participants (n)	Mortality (albumin)	Mortality (standard care without albumin)	*p* value	Heterogeneity (*I* ^2^)	Interpretation
Primary analysis (all studies)	10	11,204	23.8%	24.2%	> 0.05	0%	No significant difference
Sensitivity analysis (≤ 90 days follow‐up)	Not reported	Not reported	Similar to primary analysis	Similar to primary analysis	> 0.05	Lower than primary	Results remained consistent

These findings support the robustness of the primary analysis and indicate that longer follow‐up durations did not significantly influence the overall mortality results.

In general, subgroup analyses showed consistent effects across clinical conditions, with no statistically significant interaction; however, these findings should be interpreted cautiously due to limited data and lack of power for subgroup comparisons (Appendix 3, Supporting Information).

## 4. Discussion

The administration of intravenous fluids is one of the cornerstones of resuscitation of critically ill patients; the sooner initiated, the greater the benefits [19]. The most commonly used fluids in ICUs are crystalloid solutions (saline and lactated Ringer).

### 4.1. ALB vs. Crystalloids

ALB infusion has been suggested to stabilize hemodynamic resuscitation endpoints, improve diuretic resistance, and has the potential to prevent episodes of hypotension during mechanical ventilation in hypoalbuminemic patients, with the ability to improve mortality in some situations [20].

When compared with crystalloids, no significant differences were found in mortality, transfusion requirement, or most secondary outcomes. Subgroup analysis showed that ALB could reduce the blood requirement in patients with hypovolemic shock compared to crystalloids, while increasing it in patients with septic shock.

A retrospective observational study conducted in China [21] compared the use of ALB versus crystalloids and found no significant difference in mortality between the groups. However, after adjusting for confounders, the investigators concluded that early administration of 5% ALB in combination with crystalloids could decrease mortality in patients requiring high‐volume resuscitation over a 28‐day follow‐up when compared with crystalloid solution alone. Some authors suggest that the use of ALB could reduce both the occurrence of shock and mortality [19, 20].

In line with these results, a subanalysis of the SAFE study indicated possible benefits of ALB use in patients with severe sepsis. In this case, the adjusted mortality risk was significantly lower in patients who received ALB compared to those who received saline solution (OR: 0.71; 95% CI: 0.52–0.97). However, our systematic review did not find any benefit of ALB vs. crystalloids in mortality, with a moderate certainty of the evidence.

Based on the studies cited above, several clinical practice guidelines now recommend the use of ALB for the resuscitation of patients with severe sepsis or septic shock, especially in those who do not respond adequately to crystalloids infusion [22]. In addition, a meta‐analysis in patients with sepsis concluded that resuscitation with ALB is associated with a reduction in mortality compared to the use of crystalloids. However, in patients with head trauma, ALB treatment was associated with an increase in mortality, probably due to an increase in intracranial pressure, particularly during the first week [23].

In Spain, ALB use in critically ill adults is mainly indicated in patients with liver disease. In addition, it becomes the first choice in severe sepsis or septic shock in patients who do not respond to volumes of crystalloids, though these conditions are relatively exceptional. A review carried out in Spain [24, 25] recommends ALB not to be used routinely as a resuscitation fluid, although it may be considered in certain groups of patients. However, although ALB does not appear to increase mortality in the treatment of severe sepsis and septic shock, clinical guidelines continue to favor the use of crystalloids for initial resuscitation, with a moderate certainty of evidence. In this context, the international guideline for the management of sepsis and septic shock [26] does not find a clear benefit in the use of colloidal solutions compared to crystalloids in sepsis, and the high cost of ALB reinforces the recommendation of using crystalloids for initial resuscitation.

Although some studies suggest that ALB may reduce the need for fluids in burn patients, recent meta‐analyses have not shown significant benefits in critically ill adults. An example of this is a recent meta‐analysis [27] that included 12 clinical trials comparing ALB with crystalloids on various critical illnesses and perioperative scenarios, namely, sepsis, cardiothoracic surgery, and acute brain injury. This revealed a high heterogeneity in the results of the effects of ALB. Regarding the management of burn patients, the American Burn Association (ABA) [28] suggests the use of ALB, especially after the first 12–24 h after the burn, as an option that could reduce the overall need for fluids.

In our review, although not all of these situations have been analyzed separately, no clear benefits have been observed with the use of ALB in critical adults in general, nor in any of the subgroups evaluated. Clinical guidelines recommend ALB for the resuscitation of patients with septic shock who do not respond to crystalloids, but not as the first‐choice treatment, which is in line with our results.

### 4.2. ALB vs. Colloids

The use of colloids in fluid therapy has been and continues to be a controversial issue, especially in critically ill patients. The choice of the type of fluid to be administered depends on several theoretical factors that still generate debate.

In our systematic review, when comparing ALB versus other colloids, no differences in mortality were found, although a lower blood requirement was found without a significant increase in adverse events or differences in other outcomes of interest, with a low or very low certainty of evidence.

In a review including 69 studies [29], the authors concluded that ALB is the safest colloid, while HES solutions are considered the least safe. Although ALB is significantly more expensive than other colloids and balanced solutions, the 2007 COASST study [30] demonstrated that it is cost‐effective in the treatment of severe sepsis.

A meta‐analysis by Bansal et al. [31] supports this view as it found that ALB, when compared with HES, improves survival in patients with severe sepsis at 90 days. Although data analysis is rather controversial due to the use of Bayesian methods, results suggest that ALB could be superior to HES in terms of mortality and need for renal replacement therapy. Delaney et al. [32] also found that resuscitation with ALB‐containing solutions, compared with HES, is associated with a reduction in mortality in patients with sepsis (OR: 0.82; 95% CI: 0.67–1.0).

Our findings suggest that the choice of resuscitation fluid may influence the volume of blood transfusion required, although with varying levels of certainty of the evidence. However, the lower transfusion requirement for ALB compared with other colloids (MD: −0.48, 95% CI: −0.69 to −0.26) highlights a potential benefit, although certainty is low. The evidence suggests caution in interpreting these results. Therefore, further studies are needed to clarify these findings and guide clinical decision‐making. This result corresponds to experimental studies, which suggest that ALB maintains the efficiency of plasma volume expansion even when capillary permeability is altered, and that its extravasation to the interstitial is less than that of other colloids, consequently, requiring a smaller volume to infuse [7].

Differences may be due to the fact that, in our review, some studies that compared ALB with HES were excluded because of having been retracted in the medical journals where originally published. The main reasons for retraction were malpractice, lack of approval of the clinical trial by a research ethics committee, irregularities about the informed consent of the subjects, and the absence of randomization in clinical trials that were described as randomized. Furthermore, in other cases, data falsification was confirmed in cases where no patient data had been originally recorded or no laboratory data existed to support the findings reported in the studies. It was even confirmed that, in certain studies, ALB had not been used as a comparator, even though stated otherwise. The results of these fraudulent studies had been included in numerous meta‐analyses, leading to results and conclusions not based on real data [22].

Cumulative evidence suggests that HES may be associated with serious adverse effects, such as acute kidney injury (AKI) and increased mortality in critically ill patients. The CHEST 2012 study [18], for example, demonstrated that the use of HES was associated with a higher incidence of acute renal failure and showed no clear benefit in terms of mortality compared to ALB and crystalloids.

A Cochrane systematic review [33] also concluded that the use of HES in critically ill patients significantly increased the risk of renal complications and did not provide a clear advantage over other resuscitation solutions. These findings led to a reevaluation of clinical recommendations and the retraction of several articles that had initially supported the use of HES, in an effort to protect patient safety and promote more robust evidence‐based practices. Therefore, ALB appears to be a safer colloid in the treatment of severe sepsis and septic shock.

### 4.3. ALB vs. Standard Care Without Albumin

The comparator category “no ALB treatment” refers to patients who received standard care without ALB or any additional treatment administration. In most trials, this included crystalloids or other fluids as part of routine resuscitation in both groups. Therefore, this group represents “standard fluid therapy without ALB or additional treatment” rather than the absence of treatment.

In critically ill adults, recent studies have provided important data on the use of ALB compared with standard care without ALB. A recent meta‐analysis [34] included key comparisons such as ALB vs. placebo. The authors concluded that ALB does not offer significant benefits in reducing mortality compared to placebo in the majority of critically ill patients.

A RCT [35] compared the effects of ALB vs. placebo in patients with hypovolemic shock and sepsis. Although ALB showed some advantages in hemodynamic stabilization, there was no significant reduction in mortality.

Our results indicate that the use of ALB should not be considered as a first‐line option for the majority of critically ill adults. ALB did not prove better than crystalloids or colloids. However, a slight increase in the incidence of SAEs was associated with ALB use when compared to crystalloids. No significant difference was found in the volume of blood transfusion required between ALB and crystalloids or standard care without ALB, whereas less volume was necessary with ALB compared to colloids.

Understanding the cost‐effectiveness of ALB as a resuscitation fluid is essential for a more efficient and equitable use of this resource, especially to avoid the risk of shortages. Fluid resuscitation practices have evolved, showing a preference for crystalloids, especially buffered saline solutions, rather than colloids.

International guidelines on the use of ALB in critical care recommend its use in specific situations: septic shock unresponsive to crystalloids, spontaneous bacterial peritonitis, and massive hemorrhages, but not as first‐line volume replacement in critically ill patients. Results vary by subgroup and comparison, but overall, no significant differences were found in key variables such as mortality, blood requirement, and SAEs.

Wiedermann 2023 performed a narrative rapid review of meta‐analyses on ALB infusion in critically ill and perioperative patients, emphasizing the heterogeneity of existing evidence and the lack of solid support for routine use. Our meta‐analysis, which included 35 RCTs with 13,975 participants, corroborates these findings and adds updated quantitative data demonstrating no clear mortality advantage and a higher incidence of SAEs compared to crystalloids (RR: 1.14, 95% CI: 1.01–1.29). Compared to earlier systematic reviews, our study provides three key contributions: (1) a comprehensive and updated synthesis of evidence up to 2024, excluding fraudulent data; (2) comparative analyses across different control fluids and critical care contexts; and (3) a structured evaluation of evidence quality using GRADE. Together, these findings reinforce current guideline recommendations to restrict ALB use to specific clinical indications, such as septic shock unresponsive to crystalloids, rather than as a first‐line resuscitation fluid in critically ill adults [36].

Bannard‐Smith et al. conducted a systematic review and meta‐analysis on the efficacy, safety, and effectiveness of hyperoncotic ALB solutions in patients with sepsis. They suggested a possible reduction in mortality in selected subgroups but concluded that the overall evidence was limited and heterogeneous. In line with these findings, our review found no significant mortality benefit for ALB compared with crystalloids (RR: 0.99, 95% CI: 0.93–1.05), with moderate certainty. However, unlike Bannard‐Smith et al., our review included a broader spectrum of critically ill populations (sepsis, shock, surgical, trauma, and burn patients) and multiple comparators (crystalloids, colloids, and standard care without ALB). Furthermore, we excluded retracted trials and applied the GRADE approach to assess the certainty of evidence, enhancing the methodological rigor [37]. Recently, Huang et al. analyzed the association between the ALB‐corrected anion gap (ACAG) and all‐cause mortality in ICU patients with heart failure treated with inotropes and vasopressors. They reported that elevated ACAG values were independently associated with increased mortality and improved the prognostic performance of SOFA and APS III scores. This highlights the prognostic role of ALB‐related markers, but focuses on a biomarker rather than therapeutic use. Our findings complement this evidence, showing that despite its physiological relevance, ALB administration did not confer clinical benefit in mortality or major outcomes compared to crystalloids or other colloids [38].

Li et al. found that dynamic decreases in serum ALB during hospitalization were strongly associated with inflammation, worsening nutritional status, and adverse clinical outcomes, including higher rates of infection‐related complications, longer hospital stays, and increased healthcare costs. These findings align closely with our results, supporting the notion that ALB reflects the interplay between inflammation and nutrition rather than serving solely as a nutritional marker. Together, these studies emphasize the value of monitoring ALB dynamically, particularly in older, surgical, or high‐risk patients, to guide early interventions and improve clinical and nutritional outcomes [39].

The sensitivity analysis suggests that ALB could reduce the requirement for blood and transfusions in critically ill patients, in addition to a possible decrease in the frequency of SAEs, which could improve patient safety in critical situations. However, the lack of conclusive data on mortality highlights the need for additional studies with greater methodological rigor and statistical power.

### 4.4. Clinical Implications for ED, Prehospital, and ICU Settings

Most included studies were conducted in ICU or perioperative settings; however, their findings are directly relevant to earlier phases of care, as fluid resuscitation strategies initiated in the ED or prehospital setting are typically continued during ICU management.

#### 4.4.1. ED

In the initial management of critically ill patients, early fluid resuscitation is a cornerstone of treatment. Our findings support current recommendations favoring crystalloids as the first‐line therapy, given the lack of mortality benefit associated with ALB and its higher cost. ALB may be considered in selected cases, such as septic shock unresponsive to adequate crystalloid resuscitation, but should not be routinely used as an initial strategy.

#### 4.4.2. Prehospital Care

In prehospital settings, simplicity, availability, and rapid administration are key factors. Crystalloids remain the preferred option due to ease of use and a strong safety profile. The absence of a clear benefit from ALB, together with logistical constraints (storage, cost, and preparation), limits its applicability in this context. Therefore, ALB use in prehospital care is not supported by current evidence except in highly specific and uncommon scenarios.

#### 4.4.3. ICU

In the ICU, where hemodynamic monitoring is more advanced, ALB may have a more nuanced role. Although no overall mortality benefit was observed, ALB could be considered in specific subgroups, such as patients with septic shock requiring large fluid volumes or those with hypoalbuminemia and fluid overload. In addition, the observed reduction in transfusion requirements compared to other colloids suggests a potential advantage in selected patients, although the certainty of evidence remains low.

Overall, these findings reinforce a restrictive and individualized approach to ALB use across the continuum of care. Crystalloids should remain the standard first‐line fluid in most clinical scenarios, while ALB may be reserved for specific indications guided by patient characteristics and response to initial therapy.

## 5. Limitations

Although a sensitivity analysis was performed restricted to studies with low risk of bias, approximately 30% of included studies presented unclear risk, particularly in critical areas such as blinding of participants and personnel, which casts doubts about the validity of some findings. High heterogeneity was also observed in several analyses, such as volume of fluids administered and transfusion requirements, which may make generalization of the results difficult. Therefore, the findings must be analyzed with caution.

A subgroup analysis based on ALB dose and concentration was not feasible due to substantial heterogeneity and incomplete reporting across studies. Dosing regimens varied widely in terms of total dose, timing, and duration, and ALB concentration was inconsistently reported or not extractable in a stratified manner. In addition, the concurrent use of co‐interventions such as crystalloids, vasopressors, and blood products differed between studies, introducing significant confounding. These methodological limitations, together with variability in study designs and populations, would render any dose‐ or concentration‐based subgroup analysis unreliable and at high risk of bias.

## 6. Conclusions

ALB use in critically ill adults showed no benefit in terms of mortality over crystalloids, colloids, or standard care without ALB. A lower need for volume of blood transfusion was observed with respect to colloids, though the certainty of evidence was low. SAEs were found to be higher in the ALB group when compared to crystalloids, but also with a low certainty of evidence.

Future research should focus on more practical aspects of ALB use, especially in the management of sepsis. Key questions include whether ALB can provide better hemodynamic stability compared to balanced crystalloids, its role in the prevention or treatment of AKI, and how it influences patients with septic shock and hypoalbuminemia. Furthermore, understanding the effects of ALB in patients with ARDS and its potential benefits in terms of physiological outcomes and mortality would be invaluable to clinicians. The evolving use of ALB in critically ill patients, especially in the context of the recent ALBIOS trial and ongoing studies, underscores the need for updated, evidence‐based guidelines to optimize its role in critical care settings.

## Author Contributions

Talía Gabriela Porras Suárez: study concept and design, acquisition of the data, analysis and interpretation of the data, and drafting of the manuscript.

Marta Gutiérrez Valencia: study concept and design, acquisition of the data, analysis and interpretation of the data, drafting of the manuscript, and statistical expertise.

Luis Carlos Saiz and Juan Erviti: study concept and design, acquisition of the data, drafting of the manuscript, and statistical expertise—N/A.

Juan Pedro Tirapu León, Aitor Ansotegui Hernández, and Amaia Egüés Lugea: critical revision of the manuscript for important intellectual content.

Leire Leache Alegría: study concept and design, acquisition of the data, and critical revision of the manuscript for important intellectual content.

## Funding

No funding was received for this study.

## Conflicts of Interest

The authors declare no conflicts of interest.

## Supporting Information

Additional supporting information can be found online in the Supporting Information section.

## Supporting information


**Supporting Information** Appendix and PRISMA 2020 Checklist. The Appendix provides detailed information on the PICO question, the bibliographic search strategy, and the subgroup and sensitivity analyses conducted in this study. The PICO question is outlined with specific subgroups within critically ill patients, and the bibliographic search encompasses databases such as PubMed, Embase, and Cochrane. In addition, the Appendix includes detailed subgroup analyses based on different interventions and types of shock. The PRISMA 2020 Checklist outlines the reporting guidelines followed to ensure transparency and reproducibility in the methodology and findings of this systematic review.

## Data Availability

The data that support the findings of this study are available in the Supporting Information of this article.

## Appendix A List of Articles Included in the Statistical Analyses

1. Abdelmotaal AM, Abdelsalam AM, Bakry SAD, Abdel Hafiez RH, Mabrouk AR. Effect of Hydroxyethyl starch (HES) versus 5% albumin solution on intra‐abdominal pressure in severe burn patients: A prospective randomized clinical trial. Burns. 2024 Feb; 50(1): 197‐203. doi: 10.1016/j.burns.2023.06.015. Epub 2023 Jun 28. PMID: 37833147.

2. Bisgaard J, Madsen R, Dybdal LL, Lauridsen JT, Mortensen MB, Jensen AG. Goal‐directed therapy with bolus albumin 5% is not superior to bolus ringer acetate in maintaining systemic and mesenteric oxygen delivery in major upper abdominal surgery: A randomized controlled trial. Eur J Anaesthesiol. 2020 Jun; 37(6): 491‐502. doi: 10.1097/EJA.0000000000001151. PMID: 31972601.

3. Caironi P, Tognoni G, Masson S, Fumagalli R, Pesenti A, Romero M, et al.; ALBIOS Study Investigators. Albumin replacement in patients with severe sepsis or septic shock. N Engl J Med. 2014 Apr 10; 370(15): 1412‐21. doi: 10.1056/NEJMoa1305727. Epub 2014 Mar 18. PMID: 24635772.

4. Cooper AB, Cohn SM, Zhang HS, Hanna K, Stewart TE, Slutsky AS; ALBUR Investigators. Five percent albumin for adult burn shock resuscitation: lack of effect on daily multiple organ dysfunction score. Transfusion. 2006 Jan; 46(1): 80‐9. doi: 10.1111/j.1537‐2995.2005.00667.x. PMID: 16398734.

5. Cortegiani A, Grasselli G, Meessen J, Moscarelli A, Ippolito M, Turvani F, et al. Albumin replacement therapy in immunocompromised patients with sepsis ‐ Secondary analysis of the ALBIOS trial. J Crit Care. 2021 Jun; 63: 83‐91. doi: 10.1016/j.jcrc.2021.01.016. Epub 2021 Feb 9. PMID: 33636427.

6. Dolecek M, Svoboda P, Kantorová I, Scheer P, Sas I, Bíbrová J, et al. Therapeutic influence of 20% albumin versus 6% hydroxyethylstarch on extravascular lung water in septic patients: a randomized controlled trial. Hepatogastroenterology. 2009 Nov‐Dec; 56(96): 1622‐8. PMID: 20214205.

7. Dubois MJ, Orellana‐Jimenez C, Melot C, De Backer D, Berre J, et al. Albumin administration improves organ function in critically ill hypoalbuminemic patients: A prospective, randomized, controlled, pilot study. Crit Care Med. 2006 Oct; 34(10): 2536‐40. doi: 10.1097/01.CCM.0000239119.57544.0C. PMID: 16915107.

8. Ernest D, Belzberg AS, Dodek PM. Distribution of normal saline and 5% albumin infusions in cardiac surgical patients. Crit Care Med. 2001 Dec; 29(12): 2299‐302. doi: 10.1097/00003246‐200112000‐00011. PMID: 11801830.

9. Huskisson L. Intravenous volume replacement: which fluid and why? Arch Dis Child. 1992 May; 67(5): 649‐53. doi: 10.1136/adc.67.5.649. PMID: 1294071; PMCID: PMC1793695.

10. Foley EF, Borlase BC, Dzik WH, Bistrian BR, Benotti PN. Albumin supplementation in the critically ill. A prospective, randomized trial. Arch Surg. 1990 Jun; 125(6): 739‐42. doi: 10.1001/archsurg.1990.01410180063012. PMID: 2111981.

11. Golub R, Sorrento JJ Jr, Cantu R Jr, Nierman DM, Moideen A, Stein HD. Efficacy of albumin supplementation in the surgical intensive care unit: a prospective, randomized study. Crit Care Med. 1994 Apr; 22(4): 613‐9. doi: 10.1097/00003246‐199404000‐00017. PMID: 8143470.

12. Gondos T, Marjanek Z, Ulakcsai Z, Szabó Z, Bogár L, Károlyi M, et al. Short‐term effectiveness of different volume replacement therapies in postoperative hypovolaemic patients. Eur J Anaesthesiol. 2010 Sep; 27(9): 794‐800. doi: 10.1097/EJA.0b013e32833b3504. PMID: 20520555.

13. Goodwin CW, Dorethy J, Lam V, Pruitt BA Jr. Randomized trial of efficacy of crystalloids and colloid resuscitation on hemodynamic response and lung water following thermal injury. Ann Surg. 1983 May; 197(5): 520‐31. doi: 10.1097/00000658‐198305000‐00004. PMID: 6342554; PMCID: PMC1353023.

14. Goslinga H, Eijzenbach V, Heuvelmans JH, van der Laan de Vries E, Melis VM, Schmid‐Schönbein H, et al. Custom‐tailored hemodilution with albumin and crystalloids in acute ischemic stroke. Stroke. 1992 Feb; 23(2): 181‐8. doi: 10.1161/01.str.23.2.181. PMID: 1561645.

15. Grundmann, R., von Lehndorff, C. Zur Indikation der postoperativen Humanalbumintherapie auf der Intensivstation‐Eine prospektiv randomisierte Studie. *Langenbecks Arch Chiv* 367, 235–246 (1986). https://doi.org/10.1007/BF01263404

16. Jelenko C 3rd, Wheeler ML, Callaway BD, Divilio LT, Bucklen KR, Holdredge TD. Shock and resuscitation. II: Volume repletion with minimal edema using the “HALFD” (Hypertonic Albuminated Fluid Demand) regimen. JACEP. 1978 Sep; 7(9): 326‐33. doi: 10.1016/s0361‐1124(78)80356‐6. PMID: 45702.

17. Johnson SD, Lucas CE, Gerrick SJ, Ledgerwood AM, Higgins RF. Altered coagulation after albumin supplements for treatment of oligemic shock. Arch Surg. 1979 Apr; 114(4): 379‐83. doi: 10.1001/archsurg.1979.01370280033005. PMID: 435053.

18. Lazrove S, Waxman K, Shippy C, Shoemaker WC. Hemodynamic, blood volume, and oxygen transport responses to albumin and hydroxyethyl starch infusions in critically ill postoperative patients. Crit Care Med. 1980 May; 8(5): 302‐6. doi: 10.1097/00003246‐198005000‐00007. PMID: 6154562.

19. Lowe RJ, Moss GS, Jilek J, Levine HD. Crystalloids vs colloid in the etiology of pulmonary failure after trauma: a randomized trial in man. Surgery. 1977 Jun; 81(6): 676‐83. PMID: 860200.

20. Lowe RJ, Moss GS, Jilek J, Levine HD. Crystalloids versus colloid in the etiology of pulmonary failure after trauma‐‐a randomized trial in man. Crit Care Med. 1979 Mar; 7(3): 107‐12. doi: 10.1097/00003246‐197903000‐00005. PMID: 436425.

21. Lucas CE, Bouwman DL, Ledgerwood AM, Higgins R. Differential serum protein changes following supplemental albumin resuscitation for hypovolemic shock. J Trauma. 1980 Jan; 20(1): 47‐51. PMID: 7351677.

22. Maiwall R, Kumar A, Pasupuleti SSR, Hidam AK, Tevethia H, Kumar G, et al. A randomized‐controlled trial comparing 20% albumin to plasmalyte in patients with cirrhosis and sepsis‐induced hypotension [ALPS trial]. J Hepatol. 2022 Sep; 77(3): 670‐682. doi: 10.1016/j.jhep.2022.03.043. Epub 2022 Apr 20. PMID: 35460725.

23. Martin GS, Moss M, Wheeler AP, Mealer M, Morris JA, Bernard GR. A randomized, controlled trial of furosemide with or without albumin in hypoproteinemic patients with acute lung injury. Crit Care Med. 2005 Aug;33(8): 1681‐7. doi: 10.1097/01.ccm.0000171539.47006.02. PMID: 16096441.

24. Oczkowski SJ, Mazzetti I, Meade MO, Hamielec C. Furosemide and albumin for diuresis of edema (FADE): a study protocol for a randomized controlled trial. Trials. 2014 Jun 12;15: 222. doi: 10.1186/1745‐6215‐15‐222. PMID: 24919684; PMCID: PMC4059098.

25. Park CHL, de Almeida JP, de Oliveira GQ, Rizk SI, Fukushima JT, Nakamura RE, et al. Lactated Ringer′s Versus 4% Albumin on Lactated Ringer′s in Early Sepsis Therapy in Cancer Patients: A Pilot Single‐Center Randomized Trial. Crit Care Med. 2019 Oct; 47(10):e798‐e805. doi: 10.1097/CCM.0000000000003900. PMID: 31356475.

26. Pesonen E, Vlasov H, Suojaranta R, Hiippala S, Schramko A, et al. Effect of 4% Albumin Solution vs Ringer Acetate on Major Adverse Events in Patients Undergoing Cardiac Surgery With Cardiopulmonary Bypass: A Randomized Clinical Trial. JAMA. 2022 Jul 19; 328(3): 251‐258. doi: 10.1001/jama.2022.10461. PMID: 35852528; PMCID: PMC9297113.

27. Philips CA, Maiwall R, Sharma MK, Jindal A, Choudhury AK, Kumar G, et al. Comparison of 5% human albumin and normal saline for fluid resuscitation in sepsis induced hypotension among patients with cirrhosis (FRISC study): a randomized controlled trial. Hepatol Int. 2021 Aug; 15(4): 983‐994. doi: 10.1007/s12072‐021‐10164‐z. Epub 2021 May 25. PMID: 34036519.

28. Rackow EC, Mecher C, Astiz ME, Griffel M, Falk JL, Weil MH. Effects of pentastarch and albumin infusion on cardiorespiratory function and coagulation in patients with severe sepsis and systemic hypoperfusion. Crit Care Med. 1989 May; 17(5): 394‐8. doi: 10.1097/00003246‐198905000‐00003. PMID: 2468447.

29. Rackow EC, Falk JL, Fein IA, Siegel JS, Packman MI, Haupt MT, et al. Fluid resuscitation in circulatory shock: a comparison of the cardiorespiratory effects of albumin, hetastarch, and saline solutions in patients with hypovolemic and septic shock. Crit Care Med. 1983 Nov; 11(11): 839‐50. PMID: 6194934.

30. Finfer S, Bellomo R, Boyce N, French J, Myburgh J, Norton R; SAFE Study Investigators. A comparison of albumin and saline for fluid resuscitation in the intensive care unit. N Engl J Med. 2004 May 27; 350(22): 2247‐56. doi: 10.1056/NEJMoa040232. PMID: 15163774

31. Stockwell MA, Scott A, Day A, Riley B, Soni N. Colloid solutions in the critically ill. A randomized comparison of albumin and polygeline 2. Serum albumin concentration and incidences of pulmonary edema and acute renal failure. Anesthesia. 1992 Jan; 47(1): 7‐9. doi: 10.1111/j.1365‐2044.1992.tb01942.x. PMID: 1536412.

32. Suzuki T, Koyama K. Open randomized trial of the effects of 6% hydroxyethyl starch 130/0.4/9 and 5% albumin on safety profile, volume efficacy, and glycocalyx degradation in hepatic and pancreatic surgery. J Anesth. 2020 Dec; 34(6): 912‐923. doi: 10.1007/s00540‐020‐02847‐y. Epub 2020 Sep 8. PMID: 32897437.

33. Veneman TF, Oude Nijhuis J, Woittiez AJ. Human albumin and starch administration in critically ill patients: a prospective randomized clinical trial. Wien Klin Wochenschr. 2004 May 31; 116(9‐10): 305‐9. doi: 10.1007/BF03040900. PMID: 15237655.

34. Waxman K, Holness R, Tominaga G, Chela P, Grimes J. Hemodynamic and oxygen transport effects of pentastarch in burn resuscitation. Ann Surg. 1989 Mar; 209(3): 341‐5. doi: 10.1097/00000658‐198903000‐00015. PMID: 2466449; PMCID: PMC1493930.

35. Weaver DW, Ledgerwood AM, Lucas CE, Higgins R, Bouwman DL, Johnson SD. Pulmonary effects of albumin resuscitation for severe hypovolemic shock. Arch Surg. 1978 Apr; 113(4): 387‐92. doi: 10.1001/archsurg.1978.01370160045006. PMID: 637709.

## References

[1] Bern M. , Sand K. M. , Nilsen J. , Sandlie I. , and Andersen J. T. , The Role of Albumin Receptors in Regulation of Albumin Homeostasis: Implications for Drug Delivery, Journal of Controlled Release: Official Journal of the Controlled Release Society. (2015) 211, 144–162, 10.1016/j.jconrel.2015.06.006. 26055641

[2] Ferrer R. , Mateu X. , Maseda E. et al., Non-Oncotic Properties of Albumin. A Multidisciplinary Vision About the Implications for Critically Ill Patients, Expert Review of Clinical Pharmacology. (February 2018) 11, no. 2, 125–137, Epub 2017 Dec 8. PMID: 29219627 10.1080/17512433.2018.1412827. 29219627

[3] Vincent J. L. , De Backer D. , and Wiedermann C. J. , Fluid Management in Sepsis: the Potential Beneficial Effects of Albumin, Journal of Critical Care. (October 2016) 35, 161–167, Epub 2016 Apr 27. PMID: 27481753 10.1016/j.jcrc.2016.04.019. 27481753

[4] Dull R. O. and Hahn R. G. , The Glycocalyx as a Permeability Barrier: Basic Science and Clinical Evidence, Critical Care. (2022) 26, no. 1, 10.1186/s13054-022-04154-2. PMC9469578 36096866

[5] Gatta A. , Verardo A. , and Bolognesi M. , Hypoalbuminemia, Internal and Emergency Medicine. (2012) 3, no. 7 Suppl, S193–S199, 10.1007/s11739-012-0802-0. 23073857

[6] Caraceni P. , Domenicali M. , Tovoli A. et al., Clinical Indications for the Albumin Use: Still a Controversial Issue, European Journal of Internal Medicine. (December 2013) 24, no. 8, 721–728, Epub 2013 Jun 20. PMID: 23790570 10.1016/j.ejim.2013.05.015. 23790570

[7] Zazzeron L. , Gattinoni L. , and Caironi P. , Role of Albumin, Starches and Gelatins Versus Crystalloids in Volume Resuscitation of Critically Ill Patients, Current Opinion in Critical Care. (October 2016) 22, no. 5, 428–436, PMID: 27467273 10.1097/MCC.0000000000000341. 27467273

[8] Mazzaferro E. M. and Edwards T. , Update on Albumin Therapy in Critical Illness, Veterinary Clinics of North America: Small Animal Practice. (November 2020) 50, no. 6, 1289–1305, Epub 2020 Aug 21. PMID: 32839002 10.1016/j.cvsm.2020.07.005. 32839002

[9] Wiedermann C. J. , Phases of Fluid Management and the Roles of Human Albumin Solution in Perioperative and Critically Ill Patients, Current Medical Research and Opinion. (December 2020) 36, no. 12, 1961–1973, Epub 2020 Nov 5. PMID: 33090028 10.1080/03007995.2020.1840970. 33090028

[10] Lewis S. R. , Pritchard M. W. , Evans D. J. et al., Colloids Versus Crystalloids for Fluid Resuscitation in Critically Ill People, Cochrane Database of Systematic Reviews. (August 2018) no. 8, PMID: 30073665; PMCID: PMC6513027 10.1002/14651858.CD000567.pub7. PMC6513027 30073665

[11] Shamseer L. , Moher D. , Clarke M. et al., PRISMA-P Group. Preferred Reporting Items for Systematic Review and meta-analysis Protocols (PRISMA-P) 2015: Elaboration and Explanation, BMJ. (January 2015) 350, Erratum in: BMJ. 2016 Jul 21;354:i4086. doi: 10.1136/bmj.i4086. PMID: 25555855 10.1136/bmj.g7647. 25555855

[12] Graves R. S. , Users’ Guides to the Medical Literature: A Manual for Evidence-Based Clinical Practice, Journal of the Medical Library Association. (October 2002) 90, no. 4.

[13] Ouzzani M. , Hammady H. , Fedorowicz Z. , and Elmagarmid A. , Rayyan—A Web and Mobile App for Systematic Reviews, Systematic Reviews. (2016) 5, no. 1, 10.1186/s13643-016-0384-4. PMC5139140 27919275

[14] European Medicines Agency , ICH E2A: Clinical Safety Data Management: Definitions and Standards for Expedited Reporting [Internet], 1995, European Medicines Agency, London.

[15] Higgins J. P. T. , Thomas J. , Chandler J. et al., Cochrane Handbook for Systematic Reviews of Interventions Version 6.5 (Updated August 2024), 2024, Cochrane.

[16] Review Manager (RevMan) , Version (5.4), The Cochrane Collaboration. (2011) Available at revman.cochrane.org.

[17] Schumemann H. , Brożek J. , Guyatt G. , and Oxman A. , GRADE Handbook, Grading of Recommendations Assessment, Development and Evaluation. (2013) Grade Working Group, https://gdt.gradepro.org/app/handbook/handbook.html.

[18] Myburgh J. A. , Finfer S. , Bellomo R. et al., CHEST Investigators; Australian and New Zealand Intensive Care Society Clinical Trials Group. Hydroxyethyl Starch or Saline for Fluid Resuscitation in Intensive Care, New England Journal of Medicine. (November 2012) 367, no. 20, 1901–1911, Epub 2012 Oct 17. Erratum in: N Engl J Med. 2016 Mar 31;374(13):1298. doi: 10.1056/NEJMx160007. PMID: 23075127 10.1056/NEJMoa1209759. 23075127

[19] Malbrain M. L. N. G. , Langer T. , Annane D. et al., Intravenous Fluid Therapy in the Perioperative and Critical Care Setting: Executive Summary of the International Fluid Academy (IFA), Annals of Intensive Care. (May 2020) 10, no. 1, PMID: 32449147; PMCID: PMC7245999 10.1186/s13613-020-00679-3. PMC7245999 32449147

[20] Itagaki Y. , Yoshida N. , Banno M. , Momosaki R. , Yamada K. , and Hayakawa M. , Efficacy of Albumin With Diuretics in Mechanically Ventilated Patients With Hypoalbuminemia: A Systematic Review and meta-analysis, Medicine (Baltimore). (September 2022) 101, no. 37, PMID: 36123902; PMCID: PMC9478283 10.1097/MD.0000000000030276. PMC9478283 36123902

[21] Yalan Q. , Yinzhou L. , Binfei T. , Yunxing C. , Wenqi H. , and An Z. , Early High-Volume Resuscitation With Crystalloids Solution Combined With Albumin Improves Survival of Critically Ill Patients: A Retrospective Analysis from MIMIC-IV Database, Burns. (May 2024) 50, no. 4, 893–902, Epub 2024 Jan 19. PMID: 38278752 10.1016/j.burns.2024.01.016. 38278752

[22] Garnacho-Montero J. , Fernández-Mondéjar E. , Ferrer-Roca R. et al., Crystalloids and Colloids in Critical Patient Resuscitation, Medicina Intensiva. (June 2015) 39, no. 5, 303–315, Epub 2015 Feb 13. PMID: 25683695 10.1016/j.medin.2014.12.007. 25683695

[23] Latour-Pérez J. , New Recommendations on the Use of Human Albumin Solutions in Patients with Severe Sepsis and Septic Shock. A Critical Evaluation of the Literature, Medicina Intensiva. (August 2013) 37, no. 6, 409–415, Epub 2013 May 8. PMID: 23664006 10.1016/j.medin.2013.03.007. 23664006

[24] Gutiérrez M. , Leache L. , Saiz L. C. , and Erviti J. , Navarrese Health Service, MAPAC, Albumin evaluation report. https://www.navarra.es/NR/rdonlyres/B392D594-8010-4121-8F7B7BA4BE674260/467723/INF_Albumina24112020.pdf.

[25] Aguirre Puig P. , Orallo Morán M. A. , Pereira Matalobos D. , and Prieto Requeijo P. , Papel Actual de la Albúmina en Cuidados Críticos [Current Role of Albumin in Critical Care], Revista Espanola de Anestesiologia y Reanimacion. (November 2014) 61, no. 9, 497–504, Epub 2014 Jun 18. PMID: 24952825 10.1016/j.redar.2014.04.016. 24952825

[26] Valera Durán M. , Surviving Sepsis Campaign: International Guidelines for Management of Sepsis and Septic Shock, Revista Electrónica AnestesiaR. (2016) 9, no. 8, 10.30445/rear.v9i8.147.

[27] Geng L. , Tian X. , Gao Z. , Mao A. , Feng L. , and He C. , Different Concentrations of Albumin Versus Crystalloids in Patients With Sepsis and Septic Shock: A Meta-Analysis of Randomized Clinical Trials, Journal of Intensive Care Medicine. (August 2023) 38, no. 8, 679–689, Epub 2023 Apr 20. PMID: 37078161 10.1177/08850666231170778. 37078161

[28] Jeschke M. G. , van Baar M. E. , Choudhry M. A. , Chung K. K. , Gibran N. S. , and Logsetty S. , Burn Injury, Nature Reviews Disease Primers. (February 2020) 6, no. 1, PMID: 32054846; PMCID: PMC7224101 10.1038/s41572-020-0145-5. PMC7224101 32054846

[29] Méndez-López I. , Uso De Fluidoterapia en la Práctica Clínica, Boletín de Información Farmacoterapéutica de Navarra. (2023) 1–26, 10.54095/bitn20233103.

[30] Callum J. , Skubas N. J. , Bathla A. et al., International Collaboration for Transfusion Medicine Guidelines Intravenous Albumin Guideline Group. Use of Intravenous Albumin: A Guideline from the International Collaboration for Transfusion Medicine Guidelines, Chest, 10.1016/j.chest.2024.02.049. PMC11317816 38447639

[31] Fisher H. , Hsu C. Y. , Vittinghoff E. , Lin F. , and Bansal N. , Comparison of Associations of Urine Protein-Creatinine Ratio Versus Albumin-Creatinine Ratio With Complications of CKD: A Cross-Sectional Analysis, American Journal of Kidney Diseases. (December 2013) 62, no. 6, 1102–1108, Epub 2013 Sep 14. PMID: 24041612; PMCID: PMC3840083 10.1053/j.ajkd.2013.07.013. 24041612 PMC3840083

[32] Delaney A. P. , Dan A. , McCaffrey J. , and Finfer S. , The Role of Albumin as a Resuscitation Fluid for Patients With Sepsis: A Systematic Review and Meta-Analysis, Critical Care Medicine. (February 2011) 39, no. 2, 386–391, PMID: 21248514 10.1097/CCM.0b013e3181ffe217. 21248514

[33] Perel P. , Roberts I. , and Ker K. , Colloids Versus Crystalloids for Fluid Resuscitation in Critically Ill Patients, Cochrane Database of Systematic Reviews. (February 2013) no. 2, Update in: Cochrane Database Syst Rev. 2018 Aug 03;8:CD000567. doi: 10.1002/14651858.CD000567.pub7. PMID: 23450531 10.1002/14651858.CD000567.pub6. 23450531

[34] Evans L. , Rhodes A. , Alhazzani W. et al., Surviving Sepsis Campaign: International Guidelines for Management of Sepsis and Septic Shock 2021, Intensive Care Medicine. (November 2021) 47, no. 11, 1181–1247, Epub 2021 Oct 2. PMID: 34599691; PMCID: PMC8486643 10.1007/s00134-021-06506-y. 34599691 PMC8486643

[35] Murtaza F. , Mathew M. , Fagbamila O. et al., Efficacy and Safety of Albumin for the Treatment of Hepatic Encephalopathy: An Updated Systematic Review and Meta-Analysis of Randomized Controlled Trials, Annals of Medicine and Surgery. (April 2024) 86, no. 6, 3416–3422, PMID: 38846811; PMCID: PMC11152777 10.1097/MS9.0000000000002039. 38846811 PMC11152777

[36] Wiedermann C. J. , Human Albumin Infusion in Critically Ill and Perioperative Patients: Narrative Rapid Review of Meta-Analyses From the Last Five Years, Journal of Clinical Medicine. (September 2023) 12, no. 18, PMID: 37762860; PMCID: PMC10532105 10.3390/jcm12185919. PMC10532105 37762860

[37] Bannard-Smith J. , Elrakhawy M. , Norman G. , Owen R. , Felton T. , and Dark P. , The Efficacy, Safety and Effectiveness of Hyperoncotic Albumin Solutions in Patients With Sepsis: A Systematic Review and meta-analysis, Journal of the Intensive Care Society. (June 2024) 25, no. 3, 308–318, PMID: 39224427; PMCID: PMC11366183 10.1177/17511437241259437. 39224427 PMC11366183

[38] Huang S. , Zhang Q. , Wei F. , Kutryk M. J. B. , and Zhang J. , Association of Albumin-Corrected Anion Gap With Mortality in ICU Patients with Heart Failure and Acute Kidney Injury: Analysis of the MIMIC-IV Database, European Journal of Medical Research. (August 2025) 30, no. 1, PMID: 40796876; PMCID: PMC12344888 10.1186/s40001-025-03035-y. PMC12344888 40796876

[39] Li Y. , Chen L. , Yang X. et al., Dynamic Association of Serum Albumin Changes With Inflammation, Nutritional Status and Clinical Outcomes: A Secondary Analysis of a Large Prospective Observational Cohort Study, European Journal of Medical Research. (July 2025) 30, no. 1, PMID: 40717075; PMCID: PMC12302892 10.1186/s40001-025-02925-5. PMC12302892 40717075

