# Supplementary material for: Efficacy and Safety of Albumin in Critically Ill Adults: A Systematic Review
Source: Emerg Med Int. 2026 Jul 20;2026:7434218. doi: 10.1155/emmi/7434218 (PMC13382780; doi:10.1155/emmi/7434218)
Supplement: Supplementary file 1 — Supporting Information Appendix and PRISMA 2020 Checklist. The Appendix provides detailed information on the PICO question, the bibliographic search strategy, and the subgroup and sensitivity analyses conducted in this study. The PICO question is outlined with specific subgroups within critically ill patients, and the bibliographic search encompasses databases such as PubMed, Embase, and Cochrane. In addition, the Appendix includes detailed subgroup analyses based on different interventions and types of shock. The PRISMA 2020 Checklist outlines the reporting guidelines followed to ensure transparency and reproducibility in the methodology and findings of this systematic review. [file EMMI-2026-7434218-s001.zip › Supplementary Material.pdf]

ANNEXES

Appendix 1: Description of the PICO question

**P (Population):**	Critical patients	
	**Secondary:** Within critical patients, different subgroups will be distinguished, among others:	· Critical patients in shock<br>· Critical patients with neurological pathology<br>· Critical patients with trauma<br>Critically ill patients with edema and hypoalbuminemia<br>. Critical surgical patients<br>. Burned
**I (intervention):**	**Albumin**	
**C (comparison)**	**Main:**<br>Non-albumin.<br>Other types of fluids. In cases where possible, will distinguish between:<br>.<br>Vasoactive<br>. Crystalloids<br>. Colloids other than albumin<br>. Placebo<br>. Standard of treatment<br>. Drug (other type)	
**O (outcomes)**	**Keys**	Mortality<br>Blood transfusion requirement<br>Serious adverse events
	**Important**	Volume of blood loss

		Organic dysfunction<br>Length of stay in ICU and length of hospital stay
	**Not important**	Requirement of vasoconstrictors<br>Non-serious adverse events<br>Total fluid volume<br>Fluid balance

Annex 2: Bibliographic search

Pubmed Search (07-02-2024)

Title/abstract	Mesh term	Publication type
Critical*		
Intensive care		
intensive therapy unit		
intensive treatment unit		
ICU		
neurocritic*		
neurointensive*		
shock*		
severe sepsis		
resurrect*		
Circulatory Collapse		
Circulatory Failure		
	intensive care units	
	Critical Illness	
	Critical Care	
	Resuscitation	
	Shock	
albumin*		
	albumins	

randomized
placebo

randomized
controlled trial

EMBASE Search 07-02-2024

No.	Query	Results
#28	#27 AND [embase]/lim NOT ([embase]/lim AND [medline]/lim)	1412
#27	#19 AND #22 AND #26	3440
#26	#23 OR #24 OR #25	2165641
#25	'double blind':ti,ab	228225
#24	placebo:ti,ab	372155
#23	random*:ti,ab	2022234
#22	#20 OR #21	323887
#21	'albumin'/exp	172233
#20	albumin*:ti,ab	264136
#19	#1 OR #2 OR #3 OR #4 OR #5 OR #6 OR #7 OR #8 OR #9 OR #10 OR #11 OR #12 OR #13 OR #14 OR #15 OR #16 OR #17 OR #18	2746011
#18	'shock'/exp	184459
#17	'resuscitation'/exp	140700
#16	'critically ill patient'/exp	68046
#15	'critical illness'/exp	36205
#14	'intensive care unit'/exp	306532
#13	'intensive care'/exp	923724
#12	'circulatory failure':ti,ab	3709
#11	'circulatory collapse':ti,ab	1716
#10	resurrect*:ti,ab	113639
#9	'severe sepsis':ti,ab	16329
#8	shock*:ti,ab	303067
#7	neurointensive*:ti,ab	1774
#6	neurocritic*:ti,ab	4023

#5	icu:ti,ab	172654
#4	'intensive treatment unit':ti,ab	162
#3	'intensive therapy unit':ti,ab	747
#2	'intensive care':ti,ab	288286
#1	critical*:ti,ab	1373230

Cochrane Search (07-02-2024)

Search Name: Albumin review in critical care

Date Run: 07/02/2024 14:26:31

Comment:

ID SearchHits

#2 (Critical* OR "Intensive care" OR "intensive therapy unit" OR "intensive treatment unit" OR ICU OR neurocritic* OR neurointensive* OR shock* OR "severe sepsis" OR resuscitate* OR "Circulatory Collapse" OR "Circulatory Failure"): ti, ab, kw (Word variations have been searched) 88100

#3 MeSH descriptor: [Intensive Care Units] explode all trees5967

#4 MeSH descriptor: [Critical Illness] explode all trees3639

#5 MeSH descriptor: [Critical Care] explode all trees3065

#6 MeSH descriptor: [Resuscitation] explode all trees7592

#7 MeSH descriptor: [Shock] explode all trees3421

#8 #2 OR #3 OR #4 OR #5 OR #6 OR #791142

#9 (albumin*): ti,ab,kw 20330

#10 MeSH descriptor: [Albumins] explode all trees10193

#11 #9 OR #1026560

#12 (randomized) :ti,ab,kw 1146965

#13 (placebo) :ti,ab,kw 372100

#14 (randomized controlled trial) :pt 0

#15 #12 OR #13 OR #141267140

#16 #8 AND #11 AND #151327

1322 TRIALS AND 5 SYSTEMATIC REVIEWS

From 1322 trials, excluding Pubmed and Embase: 236

6.4 SUBGROUP ANALYSIS:

6.4.1 According to specific treatment

6.4.1.1 Critical adults

Albumin vs Crystalloids

Figure 65. Mortality in critically ill adults, subgroup analysis: albumin vs crystalloids.

Study or Subgroup	Experimental Events	Experimental Total	Control Events	Control Total	Weight	Risk Ratio M-H, Random, 95% CI
**1.18.1 Ringer**						
Dubois MJ 2006	12	50	15	50	1.3%	0.80 [0.42, 1.53]
Gondos T 2010	14	50	14	50	1.4%	1.00 [0.53, 1.87]
Park CHL, 2019	96	180	83	180	12.7%	1.16 [0.94, 1.43]
Pesonne 2022	2	693	4	693	0.2%	0.50 [0.09, 2.72]
**Subtotal (95% CI)**		**973**		**973**	**15.6%**	**1.10 [0.91, 1.32]**
Total events	124		116			
Heterogeneity: Tau² = 0.00; Chi² = 2.13, df = 3 (P = 0.55); I² = 0%						
Test for overall effect: Z = 0.95 (P = 0.34)						
**1.18.3 otro**						
Caironi 2014	365	888	389	893	45.3%	0.94 [0.85, 1.05]
Cortegiany 2021	69	139	89	165	11.5%	0.92 [0.74, 1.15]
Maiwall 2022	29	50	31	50	5.4%	0.94 [0.68, 1.29]
Oczkowski SJW, 2018	6	24	2	21	0.3%	2.63 [0.59, 11.64]
SAFE 2004	185	603	217	615	21.2%	0.87 [0.74, 1.02]
Veneman TF, 2004	8	15	5	16	0.7%	1.71 [0.72, 4.06]
**Subtotal (95% CI)**		**1719**		**1760**	**84.4%**	**0.93 [0.86, 1.01]**
Total events	662		733			
Heterogeneity: Tau² = 0.00; Chi² = 4.50, df = 5 (P = 0.48); I² = 0%						
Test for overall effect: Z = 1.82 (P = 0.07)						
**Total (95% CI)**		**2692**		**2733**	**100.0%**	**0.95 [0.88, 1.03]**
Total events	786		849			
Heterogeneity: Tau² = 0.00; Chi² = 9.08, df = 9 (P = 0.43); I² = 1%						
Test for overall effect: Z = 1.27 (P = 0.20)						
Test for subgroup differences: Chi² = 2.51, df = 1 (P = 0.11), I² = 60.1%						

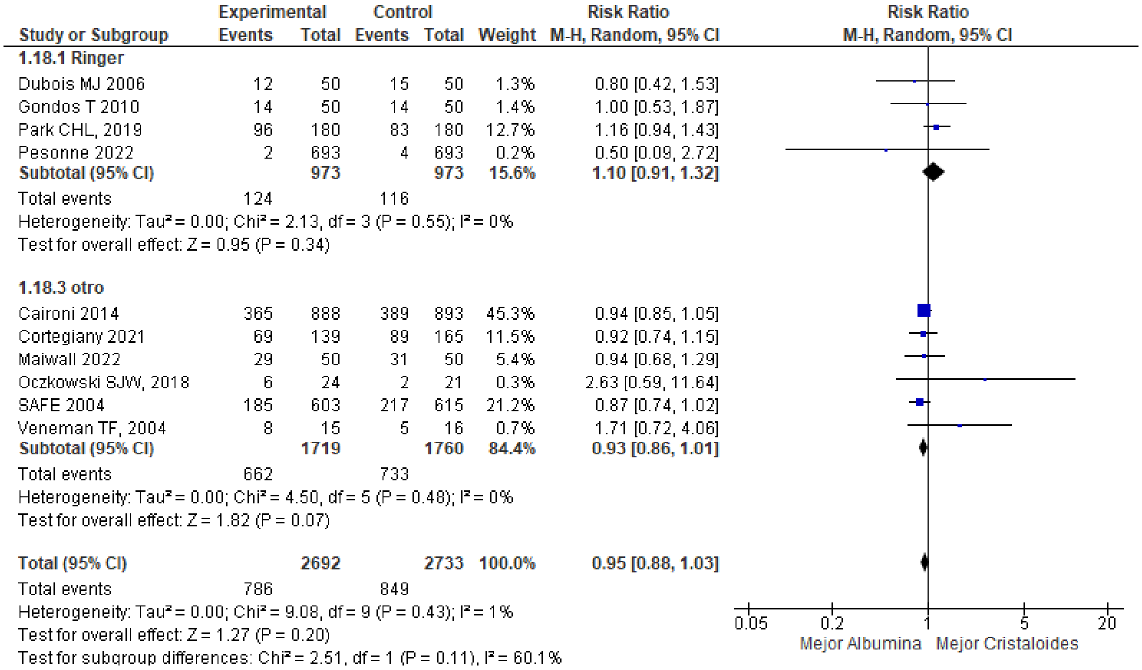

Figure 66. Blood requirement (liters) in critically ill adults, subgroup analysis: albumin vs crystalloids.

Study or Subgroup	Experimental Mean	Experimental SD	Experimental Total	Control Mean	Control SD	Control Total	Weight	Mean Difference IV, Random, 95% CI
**1.13.1 Ringer**								
Dubois MJ 2006	2.3	1.2	50	2.5	1.4	50	3.7%	-0.20 [-0.71, 0.31]
Pesonne 2022	0	0.125	693	0	0.062	693	27.6%	0.00 [-0.01, 0.01]
**Subtotal (95% CI)**			**743**			**743**	**31.3%**	**-0.00 [-0.01, 0.01]**
Heterogeneity: Tau² = 0.00; Chi² = 0.59, df = 1 (P = 0.44); I² = 0%								
Test for overall effect: Z = 0.02 (P = 0.99)								
**1.13.3 otros cristaloides**								
Caironi 2014	0.6	0.375	903	0.3	0.3	907	27.1%	0.30 [0.27, 0.33]
Rackow 1983	0.363	0.186	9	1.048	0.331	17	14.0%	-0.69 [-0.88, -0.49]
SAFE 2004	0.0436	0.167	1692	0.046	0.189	1668	27.6%	-0.00 [-0.01, 0.01]
**Subtotal (95% CI)**			**2604**			**2592**	**68.7%**	**-0.10 [-0.36, 0.17]**
Heterogeneity: Tau² = 0.05; Chi² = 362.72, df = 2 (P < 0.00001); I² = 99%								
Test for overall effect: Z = 0.72 (P = 0.47)								
**Total (95% CI)**			**3347**			**3335**	**100.0%**	**-0.02 [-0.13, 0.08]**
Heterogeneity: Tau² = 0.01; Chi² = 382.84, df = 4 (P < 0.00001); I² = 99%								
Test for overall effect: Z = 0.42 (P = 0.67)								
Test for subgroup differences: Chi² = 0.52, df = 1 (P = 0.47), I² = 0%								

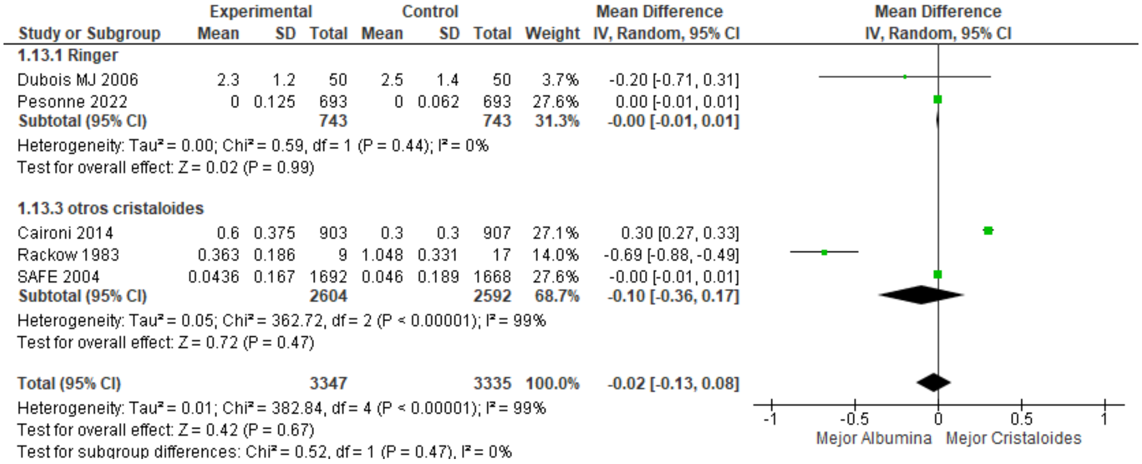

Figure 6 7. Serious adverse events in critically ill adults, subgroup analysis: albumin vs crystalloids.

Study or Subgroup	Experimental Events	Experimental Total	Control Events	Control Total	Weight	Risk Ratio M-H, Random, 95% CI
**2.3.1 Ringer**						
Dubois MJ 2006	1	50	1	50	0.2%	1.00 [0.06, 15.55]
Pesonne 2022	257	693	234	693	76.4%	1.10 [0.95, 1.27]
**Subtotal (95% CI)**		**743**		**743**	**76.6%**	**1.10 [0.95, 1.27]**
Total events	258		235			
Heterogeneity: Tau² = 0.00; Chi² = 0.00, df = 1 (P = 0.95); I² = 0%						
Test for overall effect: Z = 1.29 (P = 0.20)						
**2.3.2 otros cristaloides**						
Maiwall 2022	40	50	31	50	23.4%	1.29 [1.00, 1.67]
**Subtotal (95% CI)**		**50**		**50**	**23.4%**	**1.29 [1.00, 1.67]**
Total events	40		31			
Heterogeneity: Not applicable						
Test for overall effect: Z = 1.94 (P = 0.05)						
**Total (95% CI)**		**793**		**793**	**100.0%**	**1.14 [1.01, 1.29]**
Total events	298		266			
Heterogeneity: Tau² = 0.00; Chi² = 1.24, df = 2 (P = 0.54); I² = 0%						
Test for overall effect: Z = 2.07 (P = 0.04)						
Test for subgroup differences: Chi² = 1.16, df = 1 (P = 0.28), I² = 13.5%						

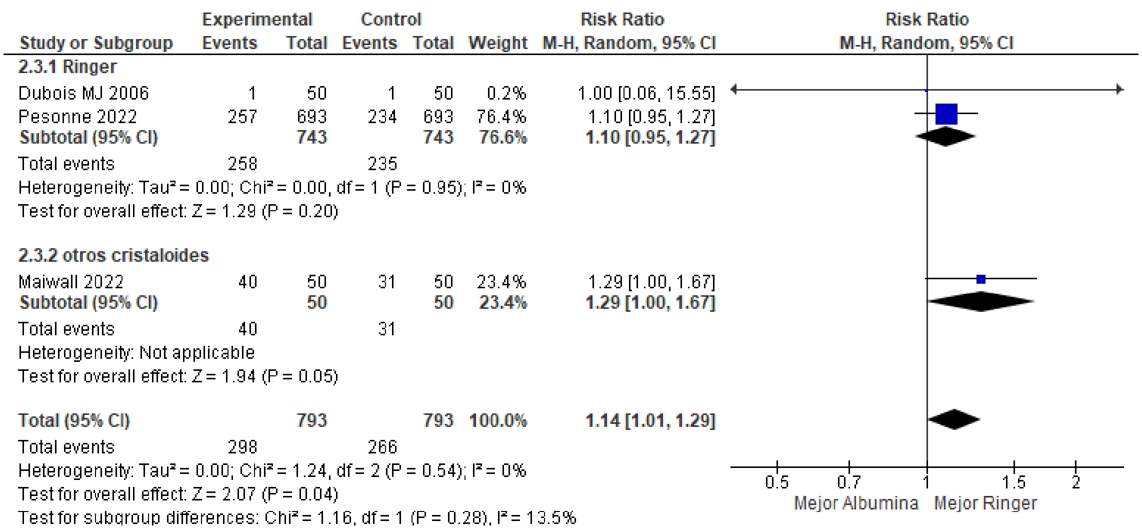

Albumin vs colloids

Figure 68. Mortality in critically ill adults, subgroup analysis: albumin vs colloids.

Study or Subgroup	Experimental Events	Experimental Total	Control Events	Control Total	Weight	Risk Ratio M-H, Random, 95% CI
**4.1.1 HES**						
Abdelmotaal AM 2024	2	26	4	26	2.3%	0.50 [0.10, 2.50]
Dolecek M 2009	4	30	6	26	4.5%	0.58 [0.18, 1.83]
Gondos T 2010	14	50	15	50	15.9%	0.93 [0.51, 1.72]
Lazrove S, 1980	3	10	3	10	3.3%	1.00 [0.26, 3.81]
Veneman TF, 2004	8	15	18	30	19.3%	0.89 [0.51, 1.55]
**Subtotal (95% CI)**		**131**		**142**	**45.4%**	**0.85 [0.59, 1.22]**
Total events	31		46			
Heterogeneity: Tau² = 0.00; Chi² = 1.04, df = 4 (P = 0.90); I² = 0%						
Test for overall effect: Z = 0.89 (P = 0.38)						
**4.1.3 otros coloides**						
Rackow EC, 1989	5	10	5	10	7.8%	1.00 [0.42, 2.40]
Stockwell MA, 1992	45	226	50	249	46.1%	0.99 [0.69, 1.42]
Waxman 1988	2	12	0	12	0.7%	5.00 [0.27, 94.34]
**Subtotal (95% CI)**		**248**		**271**	**54.6%**	**1.01 [0.73, 1.41]**
Total events	52		55			
Heterogeneity: Tau² = 0.00; Chi² = 1.16, df = 2 (P = 0.56); I² = 0%						
Test for overall effect: Z = 0.08 (P = 0.94)						
**Total (95% CI)**		**379**		**413**	**100.0%**	**0.93 [0.73, 1.19]**
Total events	83		101			
Heterogeneity: Tau² = 0.00; Chi² = 2.68, df = 7 (P = 0.91); I² = 0%						
Test for overall effect: Z = 0.54 (P = 0.59)						
Test for subgroup differences: Chi² = 0.50, df = 1 (P = 0.48), I² = 0%						

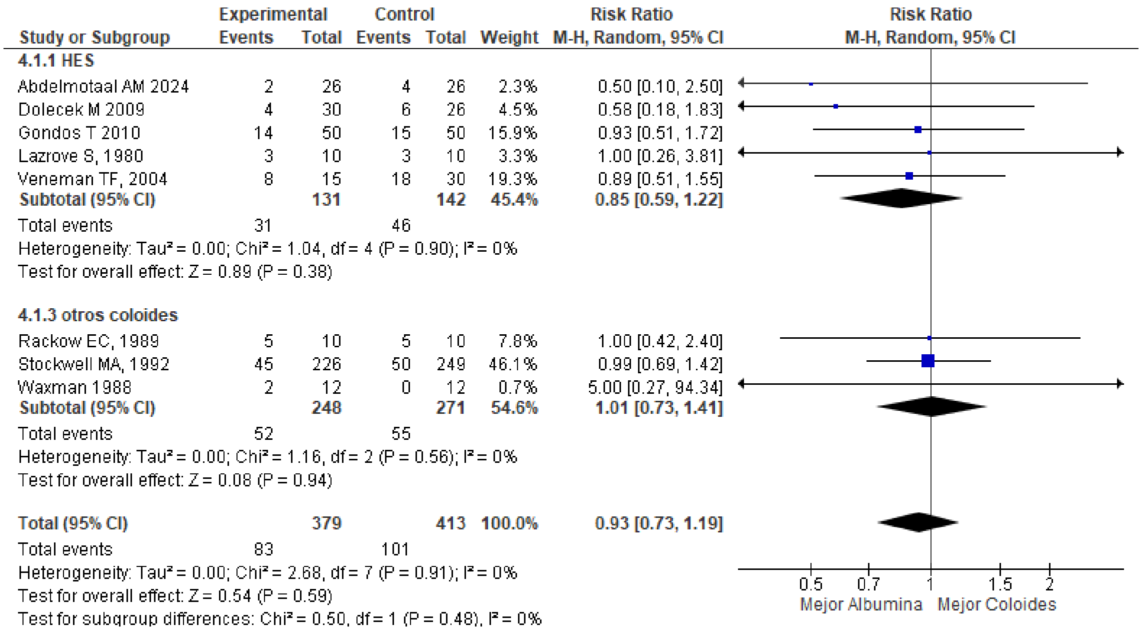

Figure 69. Serious adverse events in critically ill adults, subgroup analysis: albumin vs colloids.

Study or Subgroup	Experimental Events	Experimental Total	Control Events	Control Total	Weight	Risk Ratio M-H, Random, 95% CI
**5.2.1 HES**						
Dolecek M 2009	7	30	8	26	100.0%	0.76 [0.32, 1.81]
Lazrove S, 1980	0	10	0	10		Not estimable
**Subtotal (95% CI)**		**40**		**36**	**100.0%**	**0.76 [0.32, 1.81]**
Total events	7		8			
Heterogeneity: Not applicable						
Test for overall effect: Z = 0.62 (P = 0.53)						
**5.2.2 otros coloides**						
Waxman 1988	0	12	0	12		Not estimable
**Subtotal (95% CI)**		**12**		**12**		**Not estimable**
Total events	0		0			
Heterogeneity: Not applicable						
Test for overall effect: Not applicable						
**Total (95% CI)**		**52**		**48**	**100.0%**	**0.76 [0.32, 1.81]**
Total events	7		8			
Heterogeneity: Not applicable						
Test for overall effect: Z = 0.62 (P = 0.53)						
Test for subgroup differences: Not applicable						

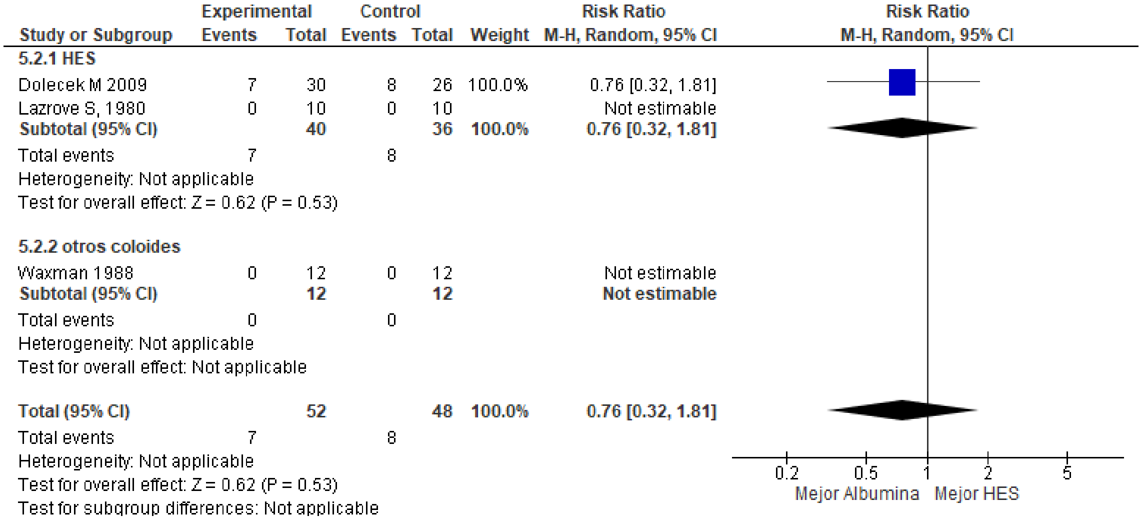

Figure 70. Blood requirement (liters) in critically ill adults, subgroup analysis: albumin vs colloids

Study or Subgroup	Experimental Mean	Experimental SD	Experimental Total	Control Mean	Control SD	Control Total	Weight	Mean Difference IV, Random, 95% CI
**4.8.1 HES**								
Falk JL 1988	0.375	0.244	6	0.7	0.288	7	26.5%	-0.32 [-0.61, -0.04]
Rackow 1983	0.363	0.186	9	0.757	0.201	9	37.8%	-0.39 [-0.57, -0.22]
**Subtotal (95% CI)**			**15**			**16**	**64.4%**	**-0.37 [-0.53, -0.22]**
Heterogeneity: Tau² = 0.00; Chi² = 0.16, df = 1 (P = 0.69); I² = 0%								
Test for overall effect: Z = 4.83 (P < 0.00001)								
**4.8.3 otro coloide**								
Rackow EC, 1989	0.369	0.186	9	1.048	0.331	17	35.6%	-0.68 [-0.88, -0.48]
**Subtotal (95% CI)**			**9**			**17**	**35.6%**	**-0.68 [-0.88, -0.48]**
Heterogeneity: Not applicable								
Test for overall effect: Z = 6.69 (P < 0.00001)								
**Total (95% CI)**			**24**			**33**	**100.0%**	**-0.48 [-0.69, -0.26]**
Heterogeneity: Tau² = 0.02; Chi² = 5.83, df = 2 (P = 0.05); I² = 66%								
Test for overall effect: Z = 4.36 (P < 0.0001)								
Test for subgroup differences: Chi² = 5.67, df = 1 (P = 0.02), I² = 82.4%								

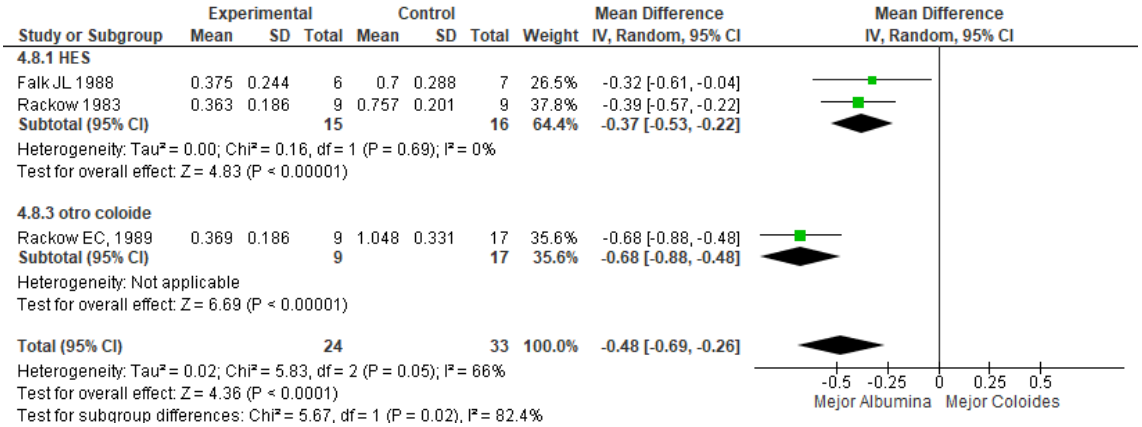

6.4.2 According to type of shock

6.4.2.1 CRITICAL ADULTS

Figure 7 5. Mortality in critically ill adults, subgroup analysis: type of shock. Albumin vs crystalloids.

Study or Subgroup	Experimental Events	Experimental Total	Control Events	Control Total	Weight	Risk Ratio M-H, Random, 95% CI
**1.18.1 Shock séptico**						
Caironi 2014	365	888	389	893	29.0%	0.94 [0.85, 1.05]
Cortegiany 2021	69	139	89	165	9.2%	0.92 [0.74, 1.15]
Maiwall 2022	29	50	31	50	4.5%	0.94 [0.68, 1.29]
Park CHL, 2019	96	180	83	180	10.1%	1.16 [0.94, 1.43]
Pesonne 2022	2	693	4	693	0.2%	0.50 [0.09, 2.72]
SAFE SS	185	603	217	615	15.8%	0.87 [0.74, 1.02]
Veneman TF, 2004	8	15	5	16	0.6%	1.71 [0.72, 4.06]
**Subtotal (95% CI)**		**2568**		**2612**	**69.5%**	**0.96 [0.87, 1.04]**
Total events	754		818			
Heterogeneity: Tau² = 0.00; Chi² = 7.01, df = 6 (P = 0.32); I² = 14%						
Test for overall effect: Z = 1.01 (P = 0.31)						
**1.18.2 Shock hipovolémico**						
Gondos T 2010	14	50	14	50	1.2%	1.00 [0.53, 1.87]
**Subtotal (95% CI)**		**50**		**50**	**1.2%**	**1.00 [0.53, 1.87]**
Total events	14		14			
Heterogeneity: Not applicable						
Test for overall effect: Z = 0.00 (P = 1.00)						
**1.18.3 otro**						
Dubois MJ 2006	12	50	15	50	1.1%	0.80 [0.42, 1.53]
Oczkowski SJW, 2018	6	24	2	21	0.2%	2.63 [0.59, 11.64]
SAFE NSS	518	2734	492	2720	27.9%	1.05 [0.94, 1.17]
**Subtotal (95% CI)**		**2808**		**2791**	**29.3%**	**1.04 [0.87, 1.24]**
Total events	536		509			
Heterogeneity: Tau² = 0.01; Chi² = 2.12, df = 2 (P = 0.35); I² = 6%						
Test for overall effect: Z = 0.44 (P = 0.66)						
**Total (95% CI)**		**5426**		**5453**	**100.0%**	**0.98 [0.91, 1.05]**
Total events	1304		1341			
Heterogeneity: Tau² = 0.00; Chi² = 11.04, df = 10 (P = 0.35); I² = 9%						
Test for overall effect: Z = 0.57 (P = 0.57)						
Test for subgroup differences: Chi² = 0.73, df = 2 (P = 0.70), I² = 0%						

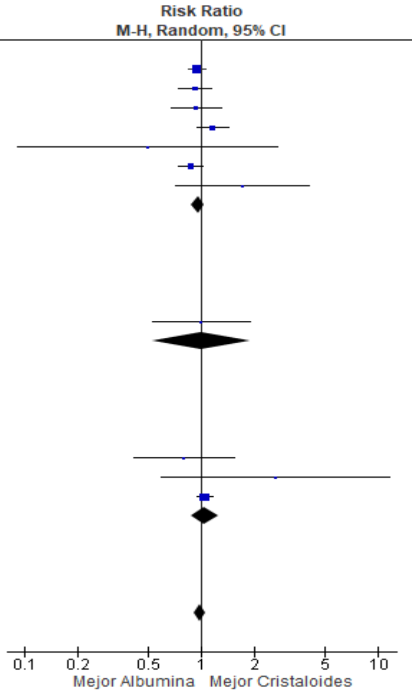

Figure 76. Serious adverse events in critically ill adults, subgroup analysis: type of shock. Albumin vs crystalloids.

Study or Subgroup	Experimental Events	Experimental Total	Control Events	Control Total	Weight	Risk Ratio M-H, Random, 95% CI
**1.3.1 Shock séptico**						
Maiwall 2022	40	50	31	50	23.4%	1.29 [1.00, 1.67]
**Subtotal (95% CI)**		**50**		**50**	**23.4%**	**1.29 [1.00, 1.67]**
Total events	40		31			
Heterogeneity: Not applicable						
Test for overall effect: Z = 1.94 (P = 0.05)						
**1.3.2 otro**						
Dubois MJ 2006	1	50	1	50	0.2%	1.00 [0.06, 15.55]
Pesonne 2022	257	693	234	693	76.4%	1.10 [0.95, 1.27]
**Subtotal (95% CI)**		**743**		**743**	**76.6%**	**1.10 [0.95, 1.27]**
Total events	258		235			
Heterogeneity: Tau² = 0.00; Chi² = 0.00, df = 1 (P = 0.95); I² = 0%						
Test for overall effect: Z = 1.29 (P = 0.20)						
**Total (95% CI)**		**793**		**793**	**100.0%**	**1.14 [1.01, 1.29]**
Total events	298		266			
Heterogeneity: Tau² = 0.00; Chi² = 1.24, df = 2 (P = 0.54); I² = 0%						
Test for overall effect: Z = 2.07 (P = 0.04)						
Test for subgroup differences: Chi² = 1.16, df = 1 (P = 0.28), I² = 13.5%						

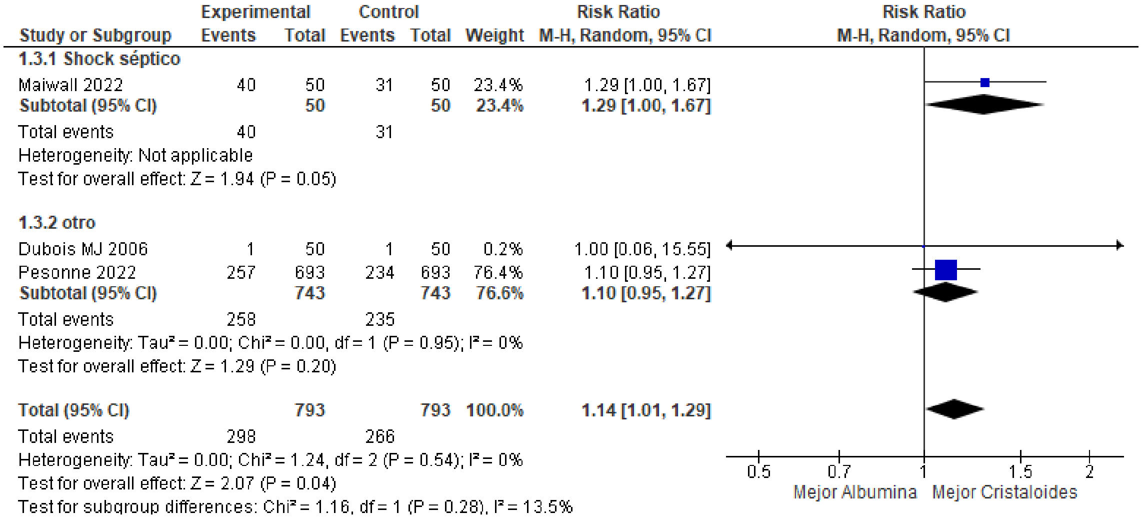

Figure 77. Blood requirement (liters) in critically ill adults, subgroup analysis: type of shock. Albumin vs crystalloids.

Study or Subgroup	Experimental Mean	Experimental SD	Experimental Total	Control Mean	Control SD	Control Total	Weight	Mean Difference IV, Random, 95% CI
**1.13.1 Shock séptico**								
Caironi 2014	0.6	0.375	903	0.3	0.3	907	27.1%	0.30 [0.27, 0.33]
**Subtotal (95% CI)**			**903**			**907**	**27.1%**	**0.30 [0.27, 0.33]**
Heterogeneity: Not applicable								
Test for overall effect: Z = 18.79 (P < 0.00001)								
**1.13.2 Shock hipovolemico**								
Rackow 1983	0.363	0.186	9	1.048	0.331	17	14.0%	-0.69 [-0.88, -0.49]
**Subtotal (95% CI)**			**9**			**17**	**14.0%**	**-0.69 [-0.88, -0.49]**
Heterogeneity: Not applicable								
Test for overall effect: Z = 6.75 (P < 0.00001)								
**1.13.3 otro**								
Dubois MJ 2006	2.3	1.2	50	2.5	1.4	50	3.7%	-0.20 [-0.71, 0.31]
Pesonne 2022	0	0.125	693	0	0.062	693	27.6%	0.00 [-0.01, 0.01]
SAFE 2004	0.0436	0.167	1692	0.046	0.189	1668	27.6%	-0.00 [-0.01, 0.01]
**Subtotal (95% CI)**			**2435**			**2411**	**58.9%**	**-0.00 [-0.01, 0.01]**
Heterogeneity: Tau^2 = 0.00; Chi^2 = 0.67, df = 2 (P = 0.72); I^2 = 0%								
Test for overall effect: Z = 0.27 (P = 0.79)								
**Total (95% CI)**			**3347**			**3335**	**100.0%**	**-0.02 [-0.13, 0.08]**
Heterogeneity: Tau^2 = 0.01; Chi^2 = 382.84, df = 4 (P < 0.00001); I^2 = 99%								
Test for overall effect: Z = 0.42 (P = 0.67)								
Test for subgroup differences: Chi^2 = 382.17, df = 2 (P < 0.00001), I^2 = 99.5%								

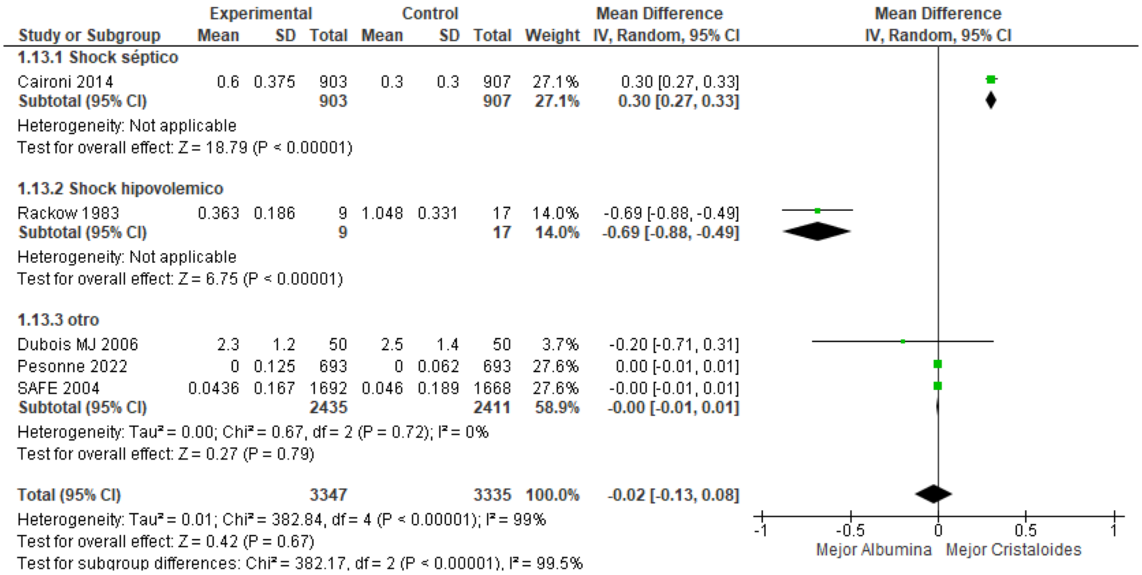

Albumin vs colloids

Figure 78. Mortality in critically ill adults, subgroup analysis: type of shock. Albumin vs colloids.

Study or Subgroup	Experimental Events	Experimental Total	Control Events	Control Total	Weight	Risk Ratio M-H, Random, 95% CI
**4.1.1 Shock séptico**						
Lazrove S, 1980	3	10	3	10	3.2%	1.00 [0.26, 3.81]
Stockwell MA, 1992	45	226	50	249	44.4%	0.99 [0.69, 1.42]
Veneman TF, 2004	8	15	18	30	18.6%	0.89 [0.51, 1.55]
Waxman 1988	2	12	0	12	0.7%	5.00 [0.27, 94.34]
**Subtotal (95% CI)**		**263**		**301**	**66.8%**	**0.98 [0.73, 1.31]**
Total events	58		71			
Heterogeneity: Tau^2 = 0.00; Chi^2 = 1.34, df = 3 (P = 0.72); I^2 = 0%						
Test for overall effect: Z = 0.15 (P = 0.88)						
**4.1.2 Shock hipovolémico**						
Gondos T 2010	14	50	27	100	19.1%	1.04 [0.60, 1.80]
**Subtotal (95% CI)**		**50**		**100**	**19.1%**	**1.04 [0.60, 1.80]**
Total events	14		27			
Heterogeneity: Not applicable						
Test for overall effect: Z = 0.13 (P = 0.90)						
**4.1.3 otro**						
Abdelmotaal AM 2024	2	26	4	26	2.2%	0.50 [0.10, 2.50]
Dolecek M 2009	4	30	6	26	4.3%	0.58 [0.18, 1.83]
Rackow EC, 1989	5	10	5	10	7.5%	1.00 [0.42, 2.40]
**Subtotal (95% CI)**		**66**		**62**	**14.1%**	**0.76 [0.40, 1.43]**
Total events	11		15			
Heterogeneity: Tau^2 = 0.00; Chi^2 = 0.92, df = 2 (P = 0.63); I^2 = 0%						
Test for overall effect: Z = 0.86 (P = 0.39)						
**Total (95% CI)**		**379**		**463**	**100.0%**	**0.95 [0.75, 1.21]**
Total events	83		113			
Heterogeneity: Tau^2 = 0.00; Chi^2 = 2.79, df = 7 (P = 0.90); I^2 = 0%						
Test for overall effect: Z = 0.39 (P = 0.70)						
Test for subgroup differences: Chi^2 = 0.62, df = 2 (P = 0.73), I^2 = 0%						

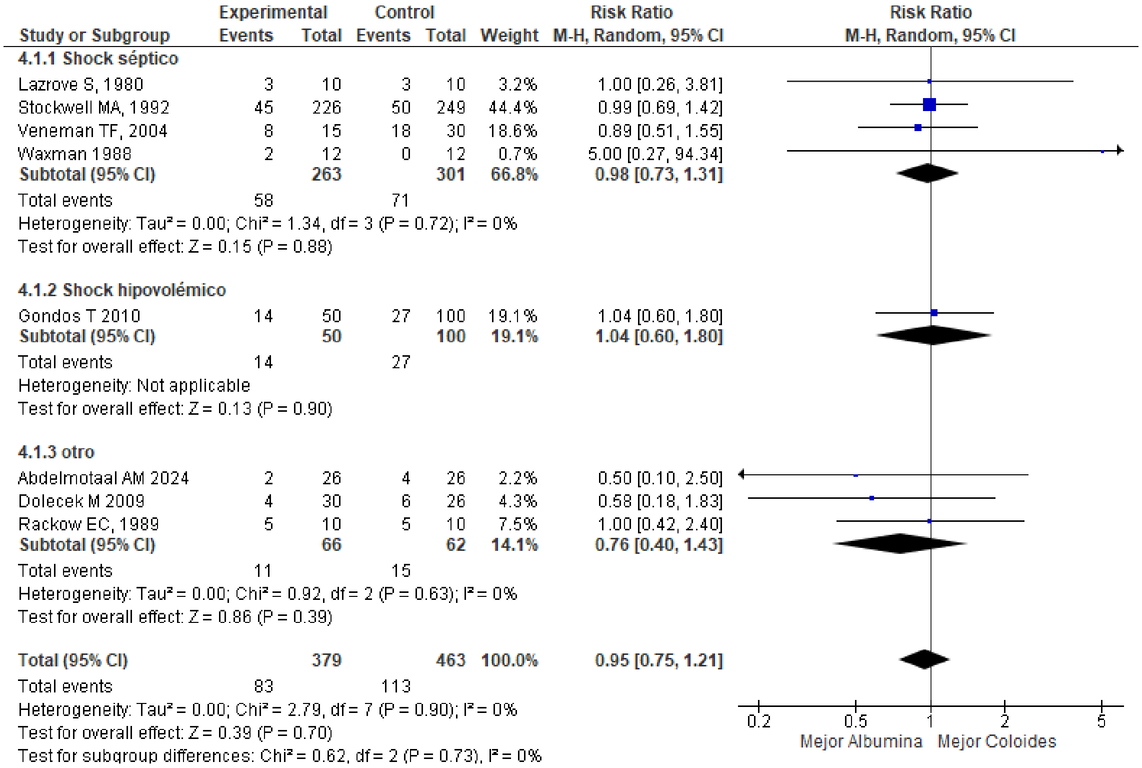

Figure 79. Blood requirement (liters) in critically ill adults, subgroup analysis: type of shock. Albumin vs colloids.

Study or Subgroup	Experimental Mean	Experimental SD	Experimental Total	Control Mean	Control SD	Control Total	Weight	Mean Difference IV, Random, 95% CI
**4.8.1 Shock séptico**								
Falk JL 1988	0.375	0.244	6	0.7	0.288	7	26.5%	-0.32 [-0.61, -0.04]
Rackow EC, 1989	0.369	0.186	9	1.048	0.331	17	35.6%	-0.68 [-0.88, -0.48]
**Subtotal (95% CI)**			**15**			**24**	**62.2%**	**-0.52 [-0.86, -0.17]**
Heterogeneity: Tau² = 0.05; Chi² = 3.91, df = 1 (P = 0.05); I² = 74%								
Test for overall effect: Z = 2.94 (P = 0.003)								
**4.8.2 Shock Hipovolémico**								
Rackow 1983	0.363	0.186	9	0.757	0.201	9	37.8%	-0.39 [-0.57, -0.22]
**Subtotal (95% CI)**			**9**			**9**	**37.8%**	**-0.39 [-0.57, -0.22]**
Heterogeneity: Not applicable								
Test for overall effect: Z = 4.32 (P < 0.0001)								
**4.8.3 otro**								
Suzuki T; 2020	0	0.08	25	0	0	25		Not estimable
**Subtotal (95% CI)**			**25**			**25**		**Not estimable**
Heterogeneity: Not applicable								
Test for overall effect: Not applicable								
**Total (95% CI)**			**49**			**58**	**100.0%**	**-0.48 [-0.69, -0.26]**
Heterogeneity: Tau² = 0.02; Chi² = 5.83, df = 2 (P = 0.05); I² = 66%								
Test for overall effect: Z = 4.36 (P < 0.0001)								
Test for subgroup differences: Chi² = 0.39, df = 1 (P = 0.53), I² = 0%								

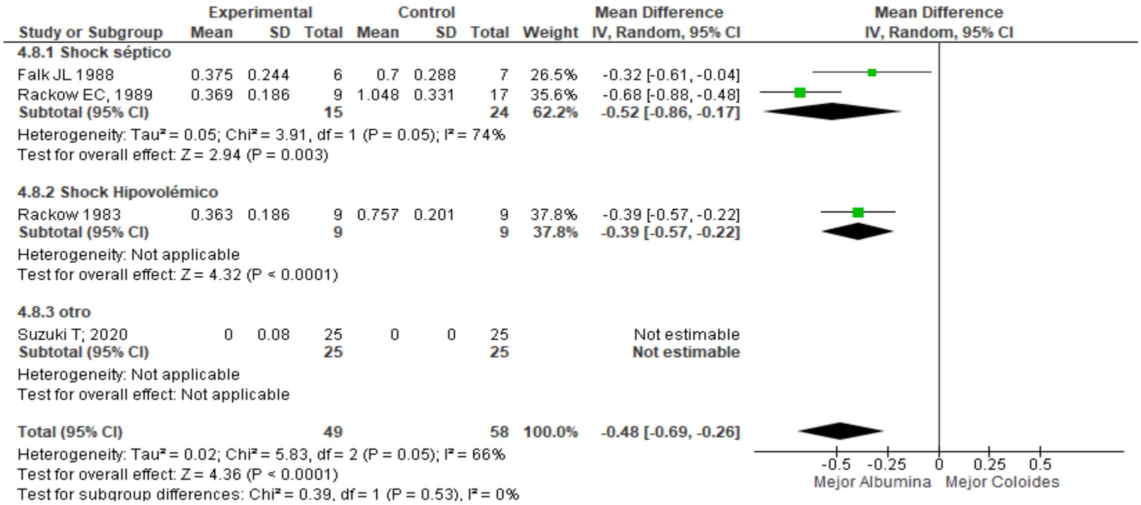

Albumin vs No treatment

Figure 80. Mortality in critically ill adults, subgroup analysis: type of shock. Albumin vs. no treatment.

Study or Subgroup	Experimental Events	Experimental Total	Control Events	Control Total	Weight	Risk Ratio M-H, Random, 95% CI
**3.1.1 Shock hipovolémico**						
Cooper AB, 2006	3	19	1	23	2.8%	3.63 [0.41, 32.13]
Weaver DW, 1978	7	27	0	25	1.7%	13.93 [0.84, 231.93]
**Subtotal (95% CI)**		**46**		**48**	**4.5%**	**6.01 [1.07, 33.69]**
Total events	10		1			
Heterogeneity: Tau² = 0.00; Chi² = 0.60, df = 1 (P = 0.44); I² = 0%						
Test for overall effect: Z = 2.04 (P = 0.04)						
**3.1.2 otro**						
Foley EF 1990	7	18	6	22	16.8%	1.43 [0.58, 3.49]
Golub R 1994	12	116	6	103	15.1%	1.78 [0.69, 4.56]
Grundmann R 1986	24	81	19	80	50.4%	1.25 [0.74, 2.09]
Jelenko C 3rd, 1978	1	7	1	5	2.1%	0.71 [0.06, 8.90]
Lowe RJ, 1977	3	55	3	82	5.5%	1.49 [0.31, 7.12]
Lowe RJ, 1979	3	57	3	84	5.5%	1.47 [0.31, 7.05]
**Subtotal (95% CI)**		**334**		**376**	**95.5%**	**1.36 [0.93, 1.98]**
Total events	50		38			
Heterogeneity: Tau² = 0.00; Chi² = 0.70, df = 5 (P = 0.98); I² = 0%						
Test for overall effect: Z = 1.61 (P = 0.11)						
**Total (95% CI)**		**380**		**424**	**100.0%**	**1.46 [1.01, 2.10]**
Total events	60		39			
Heterogeneity: Tau² = 0.00; Chi² = 4.24, df = 7 (P = 0.75); I² = 0%						
Test for overall effect: Z = 2.01 (P = 0.04)						
Test for subgroup differences: Chi² = 2.73, df = 1 (P = 0.10), I² = 63.3%						

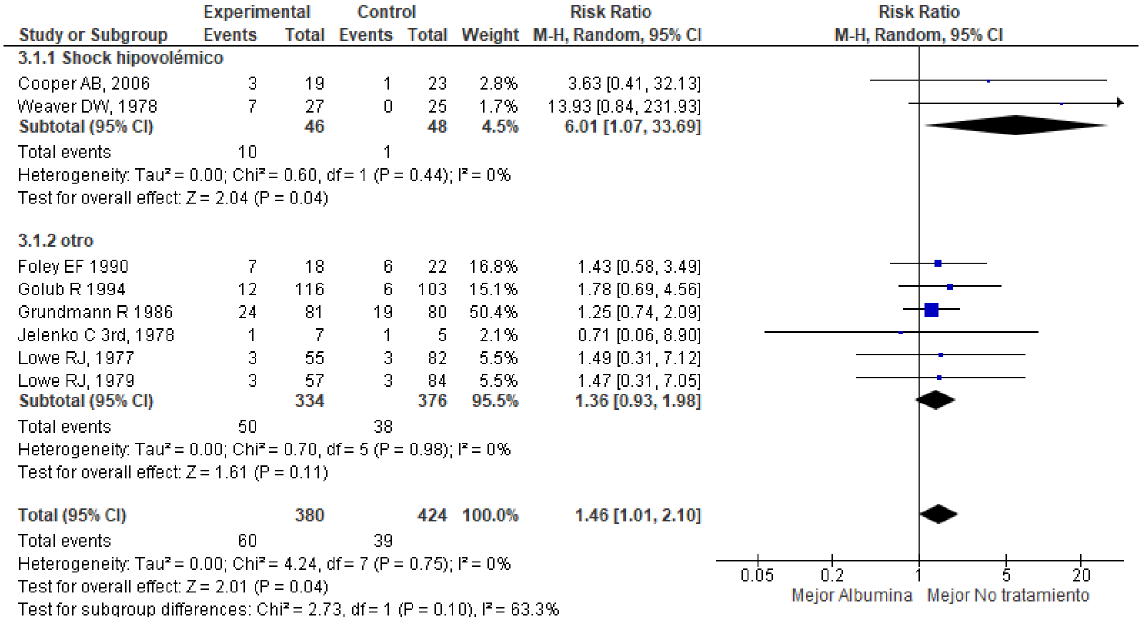

Figure 81. Blood requirement (liters) in critically ill adults, subgroup analysis: type of shock. Albumin vs. no treatment.

Study or Subgroup	Experimental Mean	Experimental SD	Experimental Total	Control Mean	Control SD	Control Total	Weight	Mean Difference IV, Random, 95% CI
**3.9.1 Shock hipovolémico**								
Johnson SD, 1979	1.52	0.466	46	1.53	0.572	48	85.2%	-0.01 [-0.22, 0.20]
**Subtotal (95% CI)**			**46**			**48**	**85.2%**	**-0.01 [-0.22, 0.20]**
Heterogeneity: Not applicable								
Test for overall effect: Z = 0.09 (P = 0.93)								
**3.9.2 otro**								
Grundmann R 1986	2.3	2.6	81	2.05	1.969	80	7.4%	0.25 [-0.46, 0.96]
Lowe RJ, 1977	1.96	3.13	55	1.5	2.87	87	3.6%	0.46 [-0.56, 1.48]
Lowe RJ, 1979	1.5	2.87	57	1.96	3.13	84	3.8%	-0.46 [-1.46, 0.54]
**Subtotal (95% CI)**			**193**			**251**	**14.8%**	**0.12 [-0.38, 0.63]**
Heterogeneity: Tau² = 0.00; Chi² = 1.84, df = 2 (P = 0.40); I² = 0%								
Test for overall effect: Z = 0.47 (P = 0.64)								
**Total (95% CI)**			**239**			**299**	**100.0%**	**0.01 [-0.18, 0.20]**
Heterogeneity: Tau² = 0.00; Chi² = 2.06, df = 3 (P = 0.56); I² = 0%								
Test for overall effect: Z = 0.09 (P = 0.92)								
Test for subgroup differences: Chi² = 0.22, df = 1 (P = 0.64), I² = 0%								

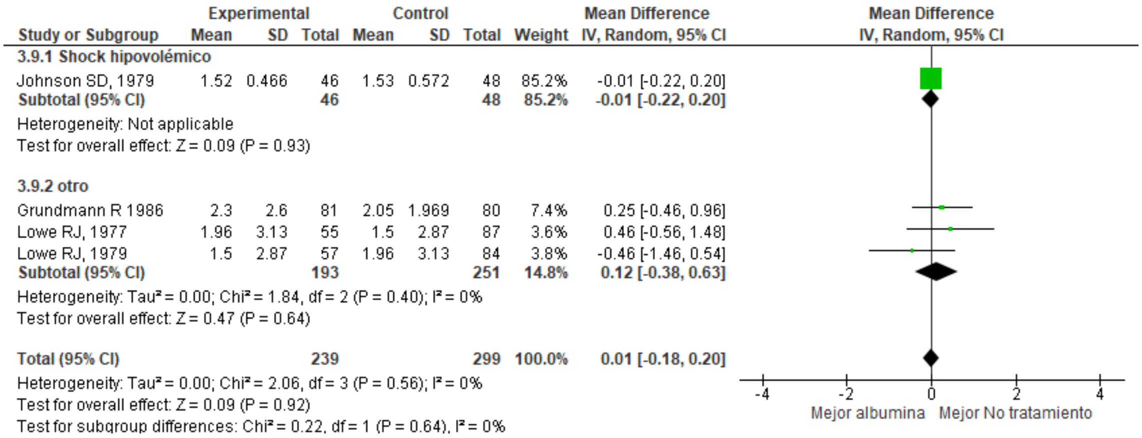

6.5 Sensitivity analysis: Studies with high risk of bias.

6.5.1 CRITICAL ADULTS

6.5.1.1 Albumin vs crystalloids

Figure 85. Mortality in critically ill adults, albumin vs crystalloids.

Study or Subgroup	Experimental Events	Experimental Total	Control Events	Control Total	Weight	Risk Ratio M-H, Random, 95% CI
Gondos T 2010	14	50	14	50	2.5%	1.00 [0.53, 1.87]
Oczkowski SJW, 2018	6	24	2	21	0.5%	2.63 [0.59, 11.64]
Park CHL, 2019	96	180	83	180	20.6%	1.16 [0.94, 1.43]
Pesonne 2022	2	693	4	693	0.3%	0.50 [0.09, 2.72]
SAFE 2004	726	3497	729	3500	74.8%	1.00 [0.91, 1.09]
Veneman TF, 2004	8	15	5	16	1.3%	1.71 [0.72, 4.06]
**Total (95% CI)**		**4459**		**4460**	**100.0%**	**1.04 [0.94, 1.15]**
Total events	852		837			
Heterogeneity: Tau² = 0.00; Chi² = 5.23, df = 5 (P = 0.39); I² = 4%						
Test for overall effect: Z = 0.72 (P = 0.47)						

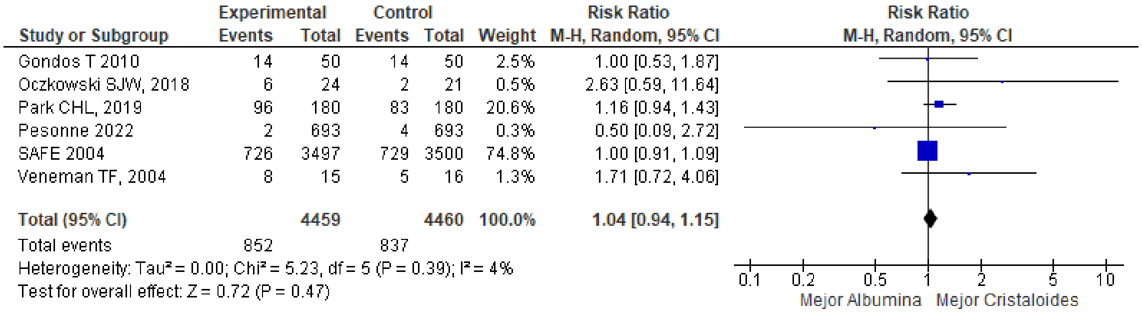

Figure 86. Blood requirement (liters) in critically ill adults, albumin vs crystalloids.

Study or Subgroup	Experimental Mean	Experimental SD	Experimental Total	Control Mean	Control SD	Control Total	Weight	Mean Difference IV, Random, 95% CI
Pesonne 2022	0	0.125	693	0	0.062	693	47.2%	0.00 [-0.01, 0.01]
Rackow 1983	0.363	0.186	9	1.048	0.331	17	5.9%	-0.69 [-0.88, -0.49]
SAFE 2004	0.0436	0.167	1692	0.046	0.189	1668	46.9%	-0.00 [-0.01, 0.01]
**Total (95% CI)**			**2394**			**2378**	**100.0%**	**-0.04 [-0.09, 0.01]**
Heterogeneity: Tau² = 0.00; Chi² = 45.49, df = 2 (P < 0.00001); I² = 96%								
Test for overall effect: Z = 1.58 (P = 0.12)								

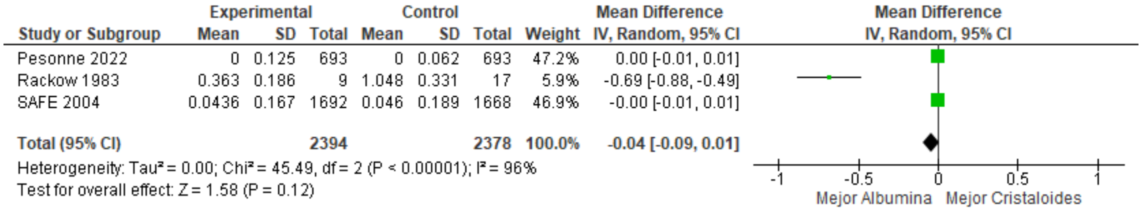

6.5.1.2 Albumin vs colloids

Figure 87. Mortality in critically ill adults, albumin vs colloids.

Study or Subgroup	Experimental Events	Experimental Total	Control Events	Control Total	Weight	Risk Ratio M-H, Random, 95% CI
Abdelmotaal AM 2024	2	26	4	26	2.2%	0.50 [0.10, 2.50]
Gondos T 2010	14	50	27	100	19.1%	1.04 [0.60, 1.80]
Lazrove S, 1980	3	10	3	10	3.2%	1.00 [0.26, 3.81]
Rackow EC, 1989	5	10	5	10	7.5%	1.00 [0.42, 2.40]
Stockwell MA, 1992	45	226	50	249	44.4%	0.99 [0.69, 1.42]
Veneman TF, 2004	8	15	18	30	18.6%	0.89 [0.51, 1.55]
Waxman 1988	2	12	0	12	0.7%	5.00 [0.27, 94.34]
**Total (95% CI)**		**379**		**463**	**100.0%**	**0.95 [0.75, 1.21]**
Total events	83		113			

Heterogeneity: Tau² = 0.00; Chi² = 2.79, df = 7 (P = 0.90); I² = 0%
Test for overall effect: Z = 0.39 (P = 0.70)

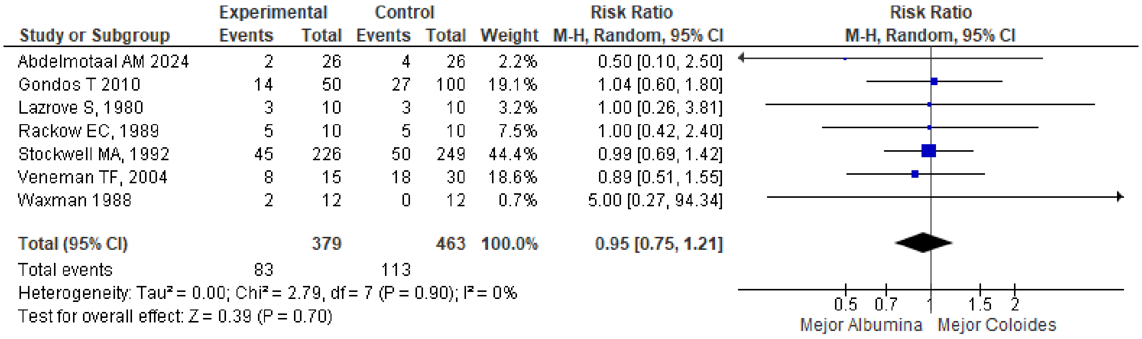

Figure 88. Blood requirement (liters) in critical adults, albumin vs colloids.

Study or Subgroup	Experimental Mean	Experimental SD	Experimental Total	Control Mean	Control SD	Control Total	Weight	Mean Difference IV, Random, 95% CI
Falk JL 1988	0.375	0.244	6	0.7	0.288	7	26.5%	-0.32 [-0.61, -0.04]
Rackow 1983	0.363	0.186	9	0.757	0.201	9	37.8%	-0.39 [-0.57, -0.22]
Rackow EC, 1989	0.369	0.186	9	1.048	0.331	17	35.6%	-0.68 [-0.88, -0.48]
**Total (95% CI)**			**24**			**33**	**100.0%**	**-0.48 [-0.69, -0.26]**

Heterogeneity: Tau² = 0.02; Chi² = 5.83, df = 2 (P = 0.05); I² = 66%
Test for overall effect: Z = 4.36 (P < 0.0001)

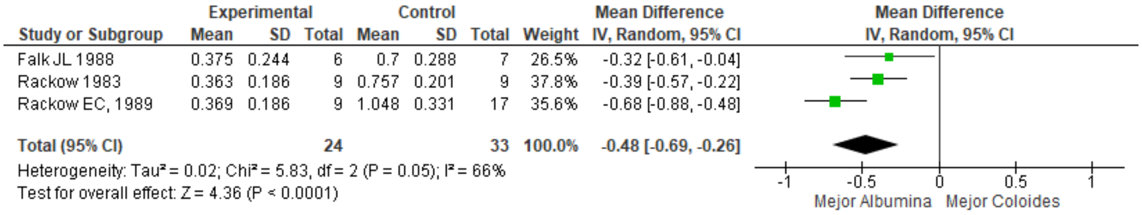

6.5.1.3 Albumin vs No treatment

Figure 89. Mortality in critically ill adults, albumin vs no treatment.

Study or Subgroup	Experimental Events	Experimental Total	Control Events	Control Total	Weight	Risk Ratio M-H, Random, 95% CI
Cooper AB, 2006	3	19	1	23	3.3%	3.63 [0.41, 32.13]
Foley EF 1990	7	18	6	22	19.3%	1.43 [0.58, 3.49]
Golub R 1994	12	116	6	103	17.4%	1.78 [0.69, 4.56]
Grundmann R 1986	24	81	19	80	58.0%	1.25 [0.74, 2.09]
Jelenko C 3rd, 1978	0	0	0	0		Not estimable
Weaver DW, 1978	7	27	0	25	2.0%	13.93 [0.84, 231.93]
**Total (95% CI)**		**261**		**253**	**100.0%**	**1.48 [1.00, 2.19]**
Total events	53		32			

Heterogeneity: Tau² = 0.00; Chi² = 3.97, df = 4 (P = 0.41); I² = 0%
Test for overall effect: Z = 1.94 (P = 0.05)

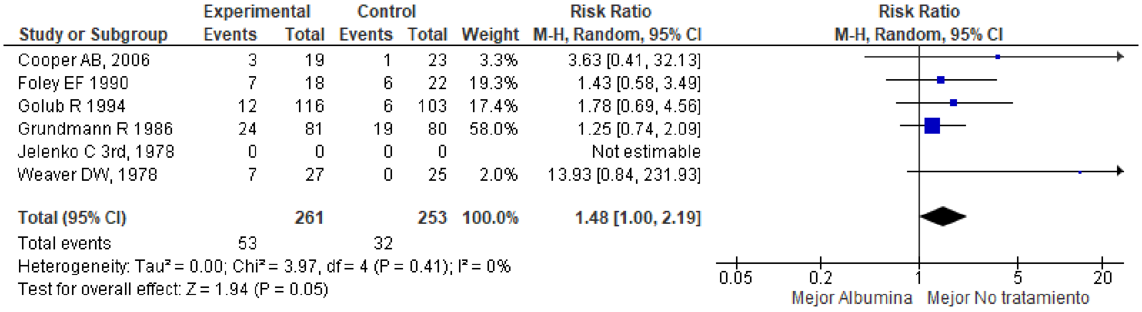

Figure 90. Blood requirement (liters) in critically ill adults, albumin vs no treatment.

Study or Subgroup	Experimental Mean	Experimental SD	Experimental Total	Control Mean	Control SD	Control Total	Weight	Mean Difference IV, Random, 95% CI
Grundmann R 1986	2.3	2.6	81	2.05	1.969	80	8.0%	0.25 [-0.46, 0.96]
Johnson SD, 1979	1.52	0.466	46	1.53	0.572	48	92.0%	-0.01 [-0.22, 0.20]
**Total (95% CI)**			**127**			**128**	**100.0%**	**0.01 [-0.19, 0.21]**

Heterogeneity: Tau² = 0.00; Chi² = 0.47, df = 1 (P = 0.49); I² = 0%
Test for overall effect: Z = 0.11 (P = 0.92)

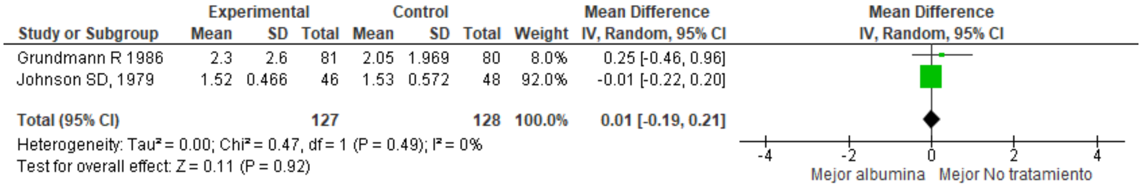

Figure 91. Serious adverse events in critically ill adults, albumin vs no treatment.

Study or Subgroup	Experimental Events	Experimental Total	Control Events	Control Total	Weight	Risk Ratio M-H, Random, 95% CI
Foley EF 1990	10	18	17	22	85.5%	0.72 [0.45, 1.15]
Grundmann R 1986	5	81	6	80	14.5%	0.82 [0.26, 2.59]
**Total (95% CI)**		**99**		**102**	**100.0%**	**0.73 [0.47, 1.13]**
Total events	15		23			

Heterogeneity: Tau² = 0.00; Chi² = 0.05, df = 1 (P = 0.82); I² = 0%
Test for overall effect: Z = 1.40 (P = 0.16)

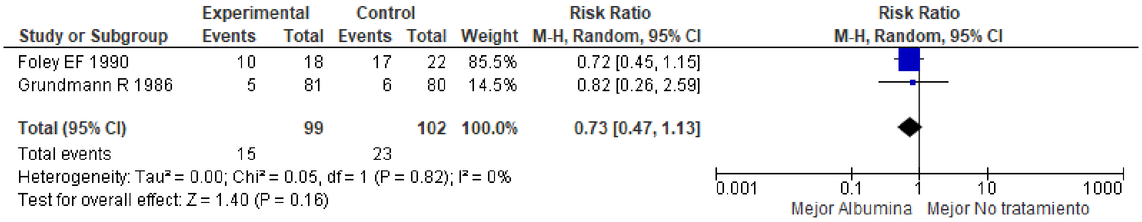

PRISMA 2020 Checklist

Section and Topic	Item #	Checklist item	Location where item is reported
**TITLE**			
Title	1	Identify the report as a systematic review.	Page 1
**ABSTRACT**			
Abstract	2	See the PRISMA 2020 for Abstracts checklist.	Page 3
**INTRODUCTION**			
Rationale	3	Describe the rationale for the review in the context of existing knowledge.	Pages 4,5
Objectives	4	Provide an explicit statement of the objective(s) or question(s) the review addresses.	Page 7
**METHODS**			
Eligibility criteria	5	Specify the inclusion and exclusion criteria for the review and how studies were grouped for the syntheses.	Page 7
Information sources	6	Specify all databases, registers, websites, organisations, reference lists and other sources searched or consulted to identify studies. Specify the date when each source was last searched or consulted.	Page 7
Search strategy	7	Present the full search strategies for all databases, registers and websites, including any filters and limits used.	Page 7
Selection process	8	Specify the methods used to decide whether a study met the inclusion criteria of the review, including how many reviewers screened each record and each report retrieved, whether they worked independently, and if applicable, details of automation tools used in the process.	Page 7, Figure 1
Data collection process	9	Specify the methods used to collect data from reports, including how many reviewers collected data from each report, whether they worked independently, any processes for obtaining or confirming data from study investigators, and if applicable, details of automation tools used in the process.	Page 8
Data items	10a	List and define all outcomes for which data were sought. Specify whether all results that were compatible with each outcome domain in each study were sought (e.g. for all measures, time points, analyses), and if not, the methods used to decide which results to collect.	Page 7
	10b	List and define all other variables for which data were sought (e.g. participant and intervention characteristics, funding sources). Describe any assumptions made about any missing or unclear information.	Page 6, 7 Table 1
Study risk of bias assessment	11	Specify the methods used to assess risk of bias in the included studies, including details of the tool(s) used, how many reviewers assessed each study and whether they worked independently, and if applicable, details of automation tools used in the process.	Page 7
Effect measures	12	Specify for each outcome the effect measure(s) (e.g. risk ratio, mean difference) used in the synthesis or presentation of results.	Page 7
Synthesis methods	13a	Describe the processes used to decide which studies were eligible for each synthesis (e.g. tabulating the study intervention characteristics and comparing against the planned groups for each synthesis (item #5)).	Page 8, Figure 1
	13b	Describe any methods required to prepare the data for presentation or synthesis, such as handling of missing summary statistics, or data conversions.	Page 8
	13c	Describe any methods used to tabulate or visually display results of individual studies and syntheses.	Page 7, 8
	13d	Describe any methods used to synthesize results and provide a rationale for the choice(s). If meta-analysis was performed, describe the model(s), method(s) to identify the presence and extent of statistical heterogeneity, and software package(s) used.	Page 7, 8
	13e	Describe any methods used to explore possible causes of heterogeneity among study results (e.g. subgroup analysis, meta-regression).	Page 8
	13f	Describe any sensitivity analyses conducted to assess robustness of the synthesized results.	Page 8
Reporting bias assessment	14	Describe any methods used to assess risk of bias due to missing results in a synthesis (arising from reporting biases).	Page 8
Certainty assessment	15	Describe any methods used to assess certainty (or confidence) in the body of evidence for an outcome.	Page 8

PRISMA 2020 Checklist

Section and Topic	Item #	Checklist item	Location where item is reported
**RESULTS**			
Study selection	16a	Describe the results of the search and selection process, from the number of records identified in the search to the number of studies included in the review, ideally using a flow diagram.	Page 8, 9
	16b	Cite studies that might appear to meet the inclusion criteria, but which were excluded, and explain why they were excluded.	Figure 1
Study characteristics	17	Cite each included study and present its characteristics.	Table 1
Risk of bias in studies	18	Present assessments of risk of bias for each included study.	Table 2
Results of individual studies	19	For all outcomes, present, for each study: (a) summary statistics for each group (where appropriate) and (b) an effect estimate and its precision (e.g. confidence/credible interval), ideally using structured tables or plots.	Table 2
Results of syntheses	20a	For each synthesis, briefly summarise the characteristics and risk of bias among contributing studies.	Table 2
	20b	Present results of all statistical syntheses conducted. If meta-analysis was done, present for each the summary estimate and its precision (e.g. confidence/credible interval) and measures of statistical heterogeneity. If comparing groups, describe the direction of the effect.	Table 2
	20c	Present results of all investigations of possible causes of heterogeneity among study results.	Table 2, Page 10-12
	20d	Present results of all sensitivity analyses conducted to assess the robustness of the synthesized results.	Supplementary material
Reporting biases	21	Present assessments of risk of bias due to missing results (arising from reporting biases) for each synthesis assessed.	Table 2, Supplementary material
Certainty of evidence	22	Present assessments of certainty (or confidence) in the body of evidence for each outcome assessed.	Table 2, Supplementary material
**DISCUSSION**			
Discussion	23a	Provide a general interpretation of the results in the context of other evidence.	Page 12-17
	23b	Discuss any limitations of the evidence included in the review.	Page 19
	23c	Discuss any limitations of the review processes used.	Page 19
	23d	Discuss implications of the results for practice, policy, and future research.	Page 19
**OTHER INFORMATION**			
Registration and protocol	24a	Provide registration information for the review, including register name and registration number, or state that the review was not registered.	Page 6
	24b	Indicate where the review protocol can be accessed, or state that a protocol was not prepared.	Page 6
	24c	Describe and explain any amendments to information provided at registration or in the protocol.	Page 6
Support	25	Describe sources of financial or non-financial support for the review, and the role of the funders or sponsors in the review.	Page 2
Competing interests	26	Declare any competing interests of review authors.	Page 2
Availability of data, code and	27	Report which of the following are publicly available and where they can be found: template data collection forms; data extracted from included studies; data used for all analyses; analytic code; any other materials used in the review.	Supplementary material

PRISMA 2020 Checklist

Section and Topic	Item #	Checklist item	Location where item is reported
other materials			

From: Page MJ, McKenzie JE, Bossuyt PM, Boutron I, Hoffmann TC, Mulrow CD, et al. The PRISMA 2020 statement: an updated guideline for reporting systematic reviews. BMJ 2021;372:n71. doi: 10.1136/bmj.n71. This work is licensed under CC BY 4.0. To view a copy of this license, visit https://creativecommons.org/licenses/by/4.0/
